# Early adulthood BMI and cardiovascular disease: a prospective cohort study from the China Kadoorie Biobank

**DOI:** 10.1016/S2468-2667(24)00043-4

**Published:** 2024-06-15

**Authors:** Yuanyuan Chen, Wei Yu, Jun Lv, Dianjianyi Sun, Pei Pei, Huaidong Du, Ling Yang, Yiping Chen, Huanxu Zhang, Junshi Chen, Zhengming Chen, Liming Li, Canqing Yu, China Kadoorie Biobank Collaborative Group

**Affiliations:** a Department of Epidemiology and Biostatistics, School of Public Health, Peking University Health Science Center, Beijing, China; b Key Laboratory of Epidemiology of Major Diseases (Peking University), Ministry of Education, Bejing, China; c Center for Public Health and Epidemic Preparedness & Response, Peking University, Beijing, China; d Clinical Trial Service Unit & Epidemiological Studies Unit, Nuffield Department of Population Health, University of Oxford, Oxford, UK; e Tongxiang Center for Disease Control and Prevention, Zhejiang, China; f China National Center for Food Safety Risk Assessment, Beijing, China

## Abstract

**Background:**

The associations of early adulthood BMI with cardiovascular diseases have yet to be completely delineated. There is little reliable evidence about these associations among east Asian populations, that differ in fat distribution, disease patterns, and lifestyle factors from other populations. We aimed to study the associations between early adulthood BMI and cardiovascular diseases in a Chinese population, and the effect of midlife lifestyle factors on outcomes.

**Methods:**

In this prospective analysis, we used data from the China Kadoorie Biobank, a large and long-term cohort from five urban areas and five rural areas, using participants aged 35–70 years. The primary outcome was the incidence of cardiovascular diseases as a group, ischaemic heart disease, haemorrhagic stroke, and ischaemic stroke, which were obtained mainly through linkage to disease registries and the national database for health insurance claims. Early adulthood BMI was assessed through self-report at baseline survey. We used Cox proportional hazards regression models to examine the prospective associations. We also undertook multiplicative and additive interaction analyses to investigate the potential modification effect of midlife healthy lifestyle factors (a combined score covering smoking, drinking, physical activity, and diet).

**Findings:**

Participants were recruited for baseline survey between June, 2004, and July, 2008. During a median follow-up of 12·0 years (IQR 11·3–13·1), we documented 57 203 (15·9%) of incident cardiovascular diseases in 360 855 participants. After adjustment for potential confounders, monotonic dose–response associations were observed between higher early adulthood BMI and increased risks of incident cardiovascular diseases. Compared with an early adulthood BMI of 20·5–22·4 kg/m^2^ (the reference group), the hazard ratios for a BMI of less than 18·5 kg/m^2^ was 0·97 (95% CI 0·94–1·00), 18·5–20·4 kg/m^2^ was 0·97 (0·95–0·99), 22·5–23·9 kg/m^2^ was 1·04 (1·02–1·07), 24·0–25·9 kg/m^2^ was 1·12 (1·09–1·15), 26·0–27·9 kg/m^2^ was 1·19 (1·14–1·24), 28·0–29·9 kg/m^2^ was 1·34 (1·25–1·44), and ≥30·0 kg/m^2^ was 1·58 (1·42–1·75). Except for haemorrhagic stroke, lower early adulthood BMI (<20·5 kg/m^2^) was associated with decreased incident cardiovascular disease risks. No significant interaction was found between midlife healthy lifestyle factors and early adulthood BMI on cardiovascular disease risks.

**Interpretation:**

Increased risks of cardiovascular disease incidence were found among participants with high early adulthood adiposity, including ischaemic heart disease, haemorrhagic stroke, and ischaemic stroke. Our findings suggest early adulthood as an important time to focus on weight management and obesity prevention for cardiovascular health later in life.

**Funding:**

National Natural Science Foundation of China, National Key Research and Development Program of China, Chinese Ministry of Science and Technology, Kadoorie Charitable Foundation, and the Wellcome Trust.

## Introduction

Much evidence has shown that obesity in middle adulthood (eg, age 35–70 years) is a well established independent risk factor for developing cardiovascular diseases and premature death in a dose–response manner.^1^ However, there is insufficient information about whether atypical weight in early adulthood (eg, age 18–34 years) could pose additional risks. Early adulthood is considered a crucial turning point for weight gain, with a continued trend of weight accumulation until middle adulthood, when weight tends to stabilise or decrease.^2^ Additionally, along with the marked increase in the prevalence rate of both overweight and obesity among adolescents over the past three decades,^3^ its prevalence in early adulthood has also been increasing globally. According to data from the China Health and Nutrition Survey,^4^ mean BMI among people aged 18–39 years has increased from 21·5 kg/m^2^ in 1993 to 23·1 kg/m^2^ in 2015, and the absolute increase in the prevalence was 12·4 percentage points for overweight and 10·3 percentage points for obesity. Thus, it is important to investigate the effect of early adulthood BMI on later health outcomes and to target this age group in obesity prevention.

Previous studies in US and European cohorts have suggested that overweight and obesity in early adulthood could be a potential risk factor for various health conditions, including all-cause mortality and cardiovascular diseases.^5^, ^6^, ^7^ However, there is a dearth of direct and high-quality evidence from Asia and low-income and middle-income countries, where the populations have distinct socioeconomic backgrounds, fat distribution, and disease patterns. Asian individuals tend to have a high proportion of body fat and prominent abdominal obesity compared with White people with similar BMI values.^8^ This difference contributes to an increased risk of developing cardiometabolic diseases at a relatively lower BMI.^9^

**Research in context**

**Evidence before this study**

We searched PubMed, EMBASE, and Google Scholar for full-length articles published from the inception of each database to May 31, 2023, with a combination of terms: (“body mass index” OR “BMI” OR “weight” OR “overweight” OR “obesity”) AND (“young” OR “early adulthood” OR “late adolescent”) AND (“lifestyle” OR “smoking” OR “tobacco use” OR “alcohol” OR “physical activity” OR “diet”). No restrictions were applied to study type or language. Relevant studies were also found by checking reference lists of identified articles. The available studies suggested that adiposity in early adulthood is an independent risk factor for developing cardiovascular diseases and premature death. However, conclusions remained uncertain and studies were primarily based on populations with European ancestry. To the best of our knowledge, only two prospective cohort studies, of unsatisfactory quality, were conducted in east Asian countries, where health determinants might differ. These studies indicated that early adulthood obesity was linked with elevated cardiovascular disease risk. In addition, despite the health benefits of healthy lifestyle factors, it is unclear whether healthy lifestyle factors in middle adulthood, a crucial modifiable influencing factor, could attenuate the adverse consequence of earlier overweight or obesity.

**Added value of this study**

With large and long-term data from a Chinese cohort, we substantiated previous findings that monotonic dose-response associations exist between higher early adulthood BMI and increased risks of incident cardiovascular diseases, which were largely independent of subsequent weight change. We also showed that excess weight in early adulthood was associated with an elevated risk of haemorrhagic stroke later in life of similar strength to the association with ischaemic stroke. In addition, our study revealed that having underweight was associated with a slightly reduced risk of ischaemic cardiovascular disease in later life. Our results showed no significant interaction between midlife healthy lifestyle factors and early adulthood BMI on the development of cardiovascular diseases, underlining the importance of weight management in young adults.

**Implications of all the available evidence**

This study expands understanding of the role of early adulthood adiposity on morbidity and mortality from cardiovascular diseases. The findings have substantial implications for public health, as they highlight the importance of targeting early adulthood as a crucial period for obesity prevention. As such, it is essential to implement relevant policies that promote weight management strategies and healthy lifestyles to empower young adults to manage their health and prevent obesity-related complications in later life.

The extent to which findings from European populations and US populations could be extrapolated to Asian populations is unclear. Moreover, the effect of underweight is still ambiguous. Some studies have found that having underweight at a young age could reduce the risks of coronary heart disease, stroke, and total cardiovascular disease.^10^ In contrast, others reported no difference between the groups with underweight and typical weight.^5^, ^6^ Furthermore, evidence is less established for haemorrhagic stroke. A meta-analysis of eight cohort studies showed an elevated risk of stroke with overweight and obesity in early adulthood.^11^ Nonetheless, this analysis was unable to clarify the effect for stroke subtypes due to few cases of haemorrhagic stroke in the existing studies, underscoring the importance of doing studies in China, where the proportion of haemorrhagic stroke is substantially higher than in most countries.^12^

Furthermore, although the benefits of healthy lifestyle factors (HLFs) are well known, few studies have investigated whether midlife HLFs could reduce the adverse effects of early adiposity for cardiovascular disease. The effects of HLFs are important as they could be a crucial modifiable infleuncing factor.

Thus, we aimed to prospectively investigate the associations of early adulthood BMI with morbidity and mortality of cardiovascular diseases with data from the Chinese Kadoorie Biobank study. We also aimed to explicitly examine whether there was effect modification through lifestyle factors in middle adulthood.

## Methods

### Study design and participants

The China Kadoorie Biobank study is a large-scale, prospective cohort with 512 725 enrolled participants aged 30–79 years from five urban areas (ie, Harbin, Qingdao, Suzhou, Liuzhou, and Haikou) and five rural areas (ie, Gansu, Henan, Sichuan, Zhejiang, and Hunan). Details of the study design and methods have been reported before, with all non-disabled, permanent residents within the age range of each region invited to participate in the study.^13^, ^14^ In brief, trained investigators carried out the baseline survey between June, 2004, and July, 2008. All participants completed an interviewer-administered questionnaire, gave a range of physical measurements, and provided written informed consent. Sex was self-reported by the participants (options were female or male). In addition to long-term follow-up, approximately 5% of surviving cohort members are randomly selected (through blocks) for resurvey from each of the ten regions every 5–6 years.

Individuals in the China Kadoorie Biobank with self-reported histories of heart disease, stroke or transient ischaemic attack, or cancer at the baseline survey were excluded, as were participants who had self-reported diabetes or screen-detected diabetes (random blood glucose ≥11·1 mmol/L or fasting glucose ≥7·0 mmol/L at baseline). We excluded these participants to minimise the possibility of reverse causality. We also excluded participants outside the age range of 35–70 years during the baseline survey and those with missing data for BMI or weight at age 25 years from the analysis.

The study protocol was approved by the Ethics Review Committee of the Chinese Centre for Disease Control and Prevention (Beijing, China; 005/2004) and the Oxford Tropical Research Ethics Committee, University of Oxford (Oxford, UK; 025-04).

### Assessment of weight and BMI

Early adulthood weight was self-reported by the participant's recall of their weight at age 25 years in the questionnaire at baseline survey. When necessary, the timepoint was widened to the time-associated events around the age of 25 years (eg, before or after graduation from university, taking a job, or marriage) or left blank if participants were unable to recall during the interview. The early adulthood weight reported in the first resurvey was similar to that in the baseline survey (intraclass correlation coefficient 0·81, 95% CI 0·80–0·81) among the 14 833 participants who attended both surveys.

Early adulthood BMI was then computed as early adulthood weight (kg) divided by height squared (m^2^). The height was measured by trained technicians at the baseline survey following standard protocols.^14^

### Measures of lifestyle factors and other covariates

Lifestyle factors assessed in this study were obtained through the questionnaire at baseline survey. We considered four midlife lifestyle factors in this study: smoking, drinking, physical activity, and diet. Smoking and drinking patterns were assessed in the questionnaire through frequency, type, and daily quantity. As described previously, total physical activity was represented by metabolic equivalent hours per day.^15^ To assess dietary patterns, we evaluated the consumption of red meat, vegetables, fruits, and eggs by measuring frequencies. An HLF score at midlife was also constructed with these four factors according to previous work.^16^ Additionally, lifestyle factors in early adulthood, including smoking and drinking, were collected by asking the starting age for these habits in the questionnaire.

Weight change was calculated by the weight difference between age 25 years and the baseline survey. Other covariates, consisting of sociodemographic variables (age, region, education, marital status), hypertension status, and family history, were also obtained through the questionnaire and physical examination at baseline. Details about the assessments of lifestyle factors and other covariates are described in the appendix (pp 2–4).

### Follow-up and outcomes

The primary outcome in this study was morbidity of cardiovascular diseases, ischaemic heart disease (ICD-10 codes I20-I25), haemorrhage stroke (ICD-10 code I61), and ischaemic stroke (ICD-10 code I63). The secondary outcomes were all-cause mortality and mortality from cardiovascular diseases. The information on cardiovascular disease mortality and morbidity was ascertained through linkage to disease and death registries, and the national database of health insurance claims.^14^ The quality and completeness of the follow-up data were checked and confirmed annually against residential records through local residential administrators, which involved monitoring the number of people who had died or were lost to follow-up each year. The onset of the study outcomes was defined as the first event (ie, disease or mortality) from the combined records of all sources mentioned. Trained clinical staff who were masked to the baseline information coded all events with the ICD-10 to ensure the quality of event coding. More details of the follow-up procedure can be found elsewhere.^14^

### Statistical analysis

Basic characteristics were described as model-based standardised means or percentages in each category of early adulthood BMI, with adjustment for age, sex, and region through either multiple linear regression or logistic regression when appropriate.^17^ For continuous variables, the standard deviations without adjustment were also reported.

We used the Chinese Criteria of Weight for Adults^18^ as the basis for BMI classification. Since our sample size was large and allowed for a more detailed investigation of the dose–response relationship between early adulthood BMI and outcomes, we divided the participants into eight BMI groups (<18·5 kg/m^2^, 18·5–20·4 kg/m^2^, 20·5–22·4 kg/m^2^ [the reference group], 22·5–23·9 kg/m^2^, 24·0–25·9 kg/m^2^, 26·0–27·9 kg/m^2^, 28·0–29·9 kg/m^2^, and ≥30·0 kg/m^2^).

The person-years of follow-up were calculated from enrolment to the earliest occurrence of one of the following events: onset of the study outcomes, death, loss to follow-up, or the global censoring date (Dec 31, 2018).

We used Cox proportional hazards regression models stratified by age (5-year interval) and the ten regions, with age as the time scale, to estimate the association between early adulthood BMI and health outcomes. We selected confounders on the basis of an established method in which the confounders are chosen from previous evidence indicating that they are, or could be, causes of the exposure, the outcome, or both.^19^ We adjusted potential confounders gradually in several steps: model 1 adjusted sex, education, marital status, hypertension status, and family history; model 2 additionally adjusted for weight change between weight at age 25 and baseline survey weight to examine the independent association of early adulthood BMI; and model 3 additionally included the four lifestyle factors (ie, smoking, alcohol consumption, physical activity, and diet). We examined the linear trend of the association through Wald tests with a newly generated variable of the median BMI in each category. We visually verified the proportional hazards assumption through log–log plots, which were found to be reasonably parallel. We also used restricted cubic splines fitted for Cox models to examine the relationship shape with four knots at the 5th, 35th, 65th, and 95th centiles of early adulthood BMI. We tested for potential non-linearity with the likelihood ratio test by comparing the model with and without the cubic spline terms.

To estimate the effect of weight change and age span on the association, we further did subgroup analyses by age group (<45 years, 45–64 years, ≥65 years) and by weight change after age 25 years (more than –2·5 kg, –2·5 kg to 2·5 kg, more than 2·5 kg). In addition, we did subgroup analysis by lifestyles, and tested the possible multiplicative and additive interactions between early adulthood BMI and midlife HLFs, to explicitly investigate the effect modification of HLFs in middle adulthood (appendix p 4).

We ran several sensitivity analyses to examine the robustness of the association based on model 3: (1) on account of the crucial role of smoking, we repeated the analyses in never or occasional smokers, considering that model adjustment for smoking might not rule out the possibility of residual confounding; (2) we did these analyses stratified by sex; (3) participants who developed cardiovascular diseases or died within 2 years of enrolment were excluded to reduce the influence of subclinical diseases; (4) participants with weight changes of more than 2·5 kg in the past year were excluded to reduce the effect of short-term fluctuation of bodyweight and subclinical diseases; (5) as hypertension is considered a mediating factor in the association between obesity and cardiovascular diseases, we further excluded it from model 3; (6) to be consistent with the HLF score, binary variables of four lifestyles were included in model 3 rather than multicategorical or continuous variables; (7) income level was also added to the model; (8) considering death as a competing event, we applied a competing risks regression model (Fine–Gray method); (9) for missing values of weight at age 25 years, we undertook imputation with information from subsequent resurveys or the mean of the age-specific (5 years strata), sex-specific, and region-specific group; and (10) as lifestyle factors in early adulthood might be confounders to the association, we further included early lifestyle factors (ie, smoking and drinking) in the regression.

We used Stata (version 15.0) and R (version 4.2.2) for analyses.

### Role of the funding source

The funders had no role in study design, data collection, data analysis, data interpretation, or writing of the report.

## Results

Of the total participants in the Biobank, we excluded 15 472 participants with heart disease, 8884 with stroke or transient ischaemic attack, 2578 with cancer, and 30 300 with diabetes at baseline survey from this analysis. After excluding participants who were outside the age range of 35–70 years (n=35 034) at baseline and those with missing data for BMI (n=1) or weight at age 25 (n=65 159), a sample size of 360 855 individuals remained for analysis (5558 participants met multiple exclusion criteria; appendix p 27). Among these participants, the mean age at baseline was 50·0 (SD 9·1) years, 153 019 (42·4%) were male and 207 836 (57·6%) were female. The mean BMI was 21·8 (SD 2·5) kg/m^2^ in early adulthood and 23·7 (3·3) kg/m^2^ in middle adulthood. 178 633 (49·5%) participants had a BMI under 24·0 kg/m^2^ at both points. 94 698 (26·2%) participants had an increase in their BMI status from underweight or normal to overweight and 22 815 (6·3%) to obese (appendix p 5). After adjusting for age, sex, and region, individuals with higher early adulthood BMI were more likely to have a lower level of education, a higher prevalence of hypertension, and middle adulthood BMI (table 1).

**Table 1:** Baseline characteristics of participants according to early adulthood BMI grouping

		**<18·5 kg/m^2^ (n=27 513; 7·6%)**	**18·5–20·4 kg/m^2^ (n=84 200; 23·3%)**	**20·5–22·4 kg/m^2^ (n=118 387; 32·8%)**	**22·5–23·9 kg/m^2^ (n=66 046; 18·3%)**	**24·0–25·9 kg/m^2^ (n=44 422; 12·3%)**	**26·0–27·9 kg/m^2^ (n=14 879; 4·1%)**	**28·0–29·9 kg/m^2^ (n=4001; 1·1%)**	**≥30·0 kg/m^2^ (n=1407; 0·4%)**	**P_trend_**
Age at baseline, years		49·3 (8·9)	48·7 (8·9)	49·5 (9·0)	50·6 (9·0)	51·7 (9·1)	52·9 (9·3)	53·5 (9·7)	53·6 (10·5)	<0·0001
Males		7983 (29·8%)	34 310 (41·5%)	56 933 (48·2%)	30 121 (45·1%)	16 855 (37·0%)	4924 (31·8%)	1387 (33·1%)	506 (34·5%)	<0·0001
Females		19 530 (70·2%)	49 890 (58·5%)	61 454 (51·8%)	35 925 (54·9%)	27 567 (63·0%)	9955 (68·2%)	2614 (66·9%)	901 (65·5%)	<0·0001
Married		25 146 (92·2%)	78 366 (92·9%)	110 787 (93·2%)	61 595 (93·1%)	40 988 (92·8%)	13 549 (92·4%)	3575 (91·4%)	1211 (89·2%)	0·058
>6 years of education		17 231 (58·4%)	52 386 (57·8%)	67 527 (55·8%)	33 598 (53·9%)	19 951 (51·2%)	6022 (49·4%)	1574 (48·2%)	580 (47·6%)	<0·0001
Prevalent hypertension		6837 (27·6%)	22 435 (28·7%)	35 809 (30·3%)	21 603 (31·2%)	15 664 (32·9%)	5884 (36·1%)	1728 (39·0%)	680 (44·2%)	<0·0001
Parental family history of cardiovascular diseases		6007 (21·9%)	17 830 (21·4%)	25 077 (21·3%)	14 281 (21·6%)	9781 (21·7%)	3336 (21·8%)	897 (21·3%)	312 (20·8%)	0·77
Regular smoker*		5659 (30·3%)	23 648 (29·7%)	40 266 (30·3%)	21 920 (30·6%)	12 828 (31·2%)	3983 (32·5%)	1113 (32·7%)	433 (35·1%)	<0·0001
Excessive alcohol drinking†		1936 (11·0%)	8022 (10·6%)	14 522 (11·1%)	8406 (11·4%)	5387 (12·1%)	1737 (12·9%)	505 (13·7%)	203 (15·7%)	<0·0001
Physical activity, MET h/day		21·6 (12·6)	21·9 (13·6)	22·4 (14·3)	22·7 (14·5)	23·0 (14·4)	23·0 (14·0)	23·0 (13·8)	22·8 (13·5)	<0·0001
Unhealthy diet‡		26 945 (98·1%)	82 522 (98·0%)	116 197 (98·1%)	64 963 (98·3%)	43 630 (98·3%)	14 605 (98·4%)	3922 (98·3%)	1381 (98·5%)	<0·0001
Middle adulthood BMI, kg/m^2^		21·9 (3·1)	22·7 (3·0)	23·6 (3·0)	24·3 (3·1)	25·0 (3·3)	25·9 (3·5)	26·8 (3·8)	27·9 (4·4)	<0·0001
Food frequency (≥4 days per week)										
	Meat	15 805 (51·2%)	46 466 (51·1%)	60 114 (50·5%)	31 151 (50·1%)	19 732 (49·3%)	6375 (49·1%)	1668 (48·8%)	611 (49·4%)	<0·0001
	Vegetables	27 215 (98·9%)	83 361 (99·0%)	117 129 (99·0%)	65 346 (99·0%)	43 963 (99·0%)	14 729 (98·9%)	3954 (98·8%)	1388 (98·5%)	0·67
	Fruit	10 117 (31·7%)	28 427 (31·6%)	35 160 (30·5%)	18 319 (29·8%)	12 056 (28·6%)	3970 (27·5%)	1083 (27·9%)	426 (30·0%)	<0·0001
	Eggs	7011 (25·3%)	21 450 (25·3%)	29 168 (24·7%)	15 707 (24·1%)	10 367 (23·4%)	3446 (22·9%)	926 (22·3%)	337 (22·8%)	<0·0001
Healthy lifestyle score§		2·1 (0·8)	2·1 (0·8)	2·1 (0·8)	2·1 (0·8)	2·1 (0·8)	2·1 (0·8)	2·1 (0·8)	2·0 (0·9)	0·29
	0 or 1	4012 (20·7%)	16 051 (20·1%)	26 501 (20·2%)	14 413 (20·2%)	8637 (20·8%)	2784 (22·2%)	793 (22·6%)	306 (24·1%)	<0·0001
	2	13 758 (47·1%)	39 556 (46·3%)	53 547 (45·7%)	29 543 (45·5%)	19 717 (44·8%)	6500 (43·4%)	1746 (43·2%)	642 (44·5%)	<0·0001
	3 or 4	9743 (32·3%)	28 593 (33·6%)	38 339 (34·1%)	22 090 (34·3%)	16 068 (34·4%)	5595 (34·4%)	1462 (34·2%)	459 (31·4%)	0·031

Data are standardised means (SD) or number of participants (standardised %), with adjustment for age, sex, and region when appropriate, through either multiple linear regression (for continuous variables) or logistic regression (for binary variables); for continuous variables, SD are reported without adjustment. MET=metabolic equivalent of task.

* Current smokers and former smokers who quit because of illness.

† Defined as those who drank ≥30 g/day of pure alcohol or former weekly (ie, ≥1 per week) drinkers.

‡ Defined as non-daily eating of vegetables, fruits, and eggs and eating red meat daily or less than weekly; participants with at least one of these components were categorised as having an unhealthy diet.

§ Healthy lifestyle scores were calculated from the number of healthy lifestyle factors (ie, smoking, drinking, physical activity, diet; 0–4); groups were merged to enhance statistical power due to low numbers in the population.

By the end date (Dec 31, 2018), 2938 (0·8%) participants were lost to follow-up. 14 706 (4·1%) participants died from other causes before cardiovascular disease outcomes. 288 946 (80·1%) participants did not have cardiovascular diseases, nor did they die before the global censoring date. During a median follow-up of 12·0 years (IQR 11·3–13·1; total person-years 4 318 187), 24 754 (6·9%) of 360 855 participants died of any causes and 6304 (1·7%) of cardiovascular diseases. There were 57 203 (15·9%) people with incident cardiovascular diseases, 29 718 (8·2%) with incident ischaemic heart disease, 5978 (1·7%) with incident haemorrhagic stroke, and 30 192 (8·4%) with incident ischaemic stroke.

**Figure 1:**
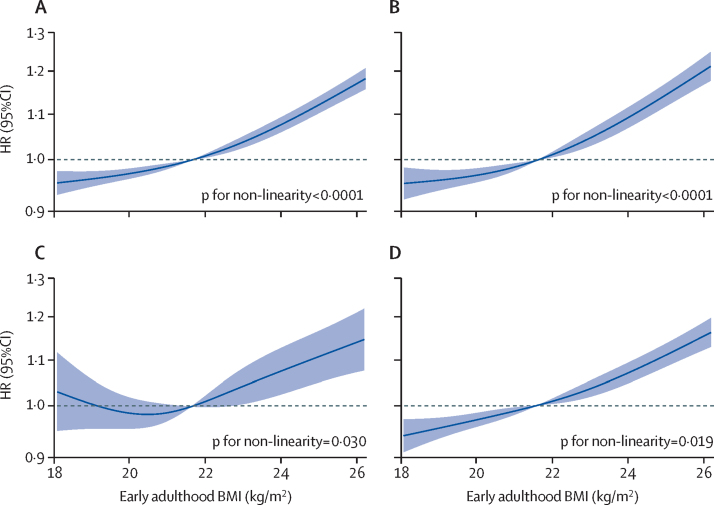
Association of early adulthood BMI with incident cardiovascular diseases Graphs show associations of early adulthood BMI with cardiovascular diseases generally (A), with ischaemic heart disease (B), with haemorrhagic stroke (C), and with ischaemic stroke (D). Estimates were adjusted for sex, education, marital status, hypertension, family history, weight change, and individual lifestyle factors, stratified by age and region. Solid lines are multivariable-adjusted HRs, with the area showing 95% CIs derived from restricted cubic spline regressions with four knots. The dashed line indicates a reference for no association at a hazard ratio of 1·0. The reference point is the median BMI (21·6 kg/m^2^). HR=hazard ratio.

After adjustment for weight change and other covariates (model 3), the risk of incident cardiovascular diseases increased monotonically with early adulthood BMI (p for non-linear trend <0·0001; figure 1). Participants with extremely high BMI (≥30·0 kg/m^2^) had a 58% higher risk of incident cardiovascular diseases than the reference group (HR 1·58; 95% CI 1·42–1·75). Obesity (BMI ≥28·0 kg/m^2^) in early adulthood was also associated with a 39% higher risk of incident cardiovascular diseases than the normal (ie, BMI of 18·5–24·0 kg/m^2^) group (HR 1·39; 95% CI 1·31–1·47; appendix pp 6–7). Similar results were found in three components of cardiovascular diseases (ischaemic heart disease, haemorrhagic stroke, and ischaemic stroke; figure 1, table 2). A higher BMI in early adulthood was associated with elevated incidence risks for cardiovascular diseases generally (p for linear trend <0·0001), ischaemic heart disease (<0·0001), haemorrhagic stroke (0·010), and ischaemic stroke (<0·0001). Additionally, having underweight was associated with a slightly reduced risk of ischaemic heart disease and ischaemic stroke (table 2).

**Table 2:** Associations of early adulthood BMI with incident cardiovascular diseases

	**<18·5 kg/m^2^ (n=27 513)**	**18·5–20·4 kg/m^2^ (n=84 200)**	**20·5–22·4 kg/m^2^ (n=118 387)**	**22·5–23·9 kg/m^2^ (n=66 046)**	**24·0–25·9 kg/m^2^ (n=44 422)**	**26·0–27·9 kg/m^2^ (n=14 879)**	**28·0–29·9 kg/m^2^ (n=4001)**	**≥30·0 kg/m^2^ (n=1407)**	P_trend_
**Cardiovascular diseases**									
Number of cases	4453	12 392	17 907	10 474	7797	2905	902	373	..
Incidence density (per 1000 person-year)	14·4 (13·98–14·83)	12·94 (12·71–13·17)	13·32 (13·13–13·52)	14·06 (13·80–14·33)	15·67 (15·33–16·02)	17·65 (17·02–18·30)	20·94 (19·62–22·35)	25·24 (22·81–27·94)	..
Model 1	1·05 (1·02–1·08)	1·00 (0·98–1·03)	1 (ref)	1·01 (0·98–1·03)	1·06 (1·03–1·09)	1·09 (1·05–1·13)	1·19 (1·11–1·27)	1·33 (1·20–1·48)	<0·0001
Model 2	0·97 (0·94–1·01)	0·97 (0·95–0·99)	1 (ref)	1·04 (1·01–1·07)	1·12 (1·09–1·15)	1·19 (1·15–1·24)	1·35 (1·26–1·44)	1·59 (1·43–1·76)	<0·0001
Model 3	0·97 (0·94–1·00)	0·97 (0·95–0·99)	1 (ref)	1·04 (1·02–1·07)	1·12 (1·09–1·15)	1·19 (1·14–1·24)	1·34 (1·25–1·44)	1·58 (1·42–1·75)	<0·0001
**Ischaemic heart disease**									
Number of cases	2454	6595	9198	5305	3986	1515	469	196	..
Incidence density (per 1000 person-year)	7·71 (7·41–8·02)	6·71 (6·55–6·87)	6·66 (6·52–6·79)	6·91 (6·73–7·10)	7·76 (7·53–8·01)	8·88 (8·44–9·34)	10·45 (9·55–11·44)	12·63 (10·98–14·53)	..
Model 1	1·07 (1·02–1·12)	1·01 (0·98–1·04)	1 (ref)	1·01 (0·97–1·04)	1·06 (1·02–1·10)	1·09 (1·04–1·16)	1·18 (1·08–1·30)	1·33 (1·16–1·54)	0·0008
Model 2	0·97 (0·92–1·01)	0·96 (0·93–0·99)	1 (ref)	1·05 (1·01–1·09)	1·15 (1·10–1·19)	1·24 (1·17–1·31)	1·40 (1·27–1·54)	1·68 (1·46–1·94)	<0·0001
Model 3	0·96 (0·92–1·01)	0·96 (0·93–0·99)	1 (ref)	1·05 (1·02–1·09)	1·15 (1·10–1·19)	1·24 (1·17–1·31)	1·40 (1·27–1·53)	1·68 (1·45–1·93)	<0·0001
**Haemorrhagic stroke**									
Number of cases	402	1126	1844	1185	914	336	110	61	..
Incidence density (per 1000 person-year)	1·23 (1·11–1·35)	1·12 (1·05–1·18)	1·30 (1·25–1·36)	1·51 (1·42–1·60)	1·73 (1·62–1·85)	1·91 (1·72–2·13)	2·36 (1·96–2·85)	3·79 (2·95–4·87)	..
Model 1	1·06 (0·95–1·18)	0·95 (0·89–1·03)	1 (ref)	1·05 (0·98–1·13)	1·14 (1·05–1·23)	1·12 (0·99–1·26)	1·24 (1·02–1·50)	1·79 (1·39–2·32)	<0·0001
Model 2	1·11 (0·99–1·24)	0·98 (0·91–1·05)	1 (ref)	1·03 (0·96–1·11)	1·09 (1·01–1·19)	1·05 (0·93–1·19)	1·15 (0·94–1·39)	1·61 (1·24–2·09)	0·021
Model 3	1·10 (0·98–1·23)	0·97 (0·90–1·05)	1 (ref)	1·03 (0·96–1·11)	1·10 (1·01–1·19)	1·06 (0·94–1·20)	1·16 (0·95–1·41)	1·61 (1·24–2·09)	0·010
**Ischaemic stroke**									
Number of cases	2282	6560	9463	5552	4128	1565	459	183	..
Incidence density (per 1000 person-year)	7·16 (6·87–7·46)	6·67 (6·51–6·84)	6·86 (6·72–6·99)	7·26 (7·07–7·45)	8·05 (7·81–8·30)	9·20 (8·76–9·67)	10·23 (9·34–11·21)	11·87 (10·27–13·72)	..
Model 1	1·03 (0·99–1·08)	1·02 (0·99–1·05)	1 (ref)	1·01 (0·98–1·04)	1·05 (1·01–1·09)	1·11 (1·05–1·17)	1·12 (1·02–1·24)	1·23 (1·06–1·42)	0·0010
Model 2	0·95 (0·91–1·00)	0·98 (0·95–1·02)	1 (ref)	1·04 (1·01– 1·08)	1·12 (1·08–1·16)	1·22 (1·15–1·29)	1·28 (1·17–1·41)	1·47 (1·27–1·71)	<0·0001
Model 3	0·95 (0·91–1·00)	0·98 (0·95–1·01)	1 (ref)	1·04 (1·01–1·08)	1·12 (1·08–1·16)	1·22 (1·15–1·29)	1·28 (1·16–1·41)	1·47 (1·26–1·70)	<0·0001

Data are n or hazard ratios (95% CI). In Model 1, hazard ratios were adjusted for sex, education, marital status, hypertension status, and family history; model 2 additionally adjusted for weight change; and model 3 additionally adjusted for four lifestyle factors (smoking, alcohol consumption, physical activity, and diet). The analyses were stratified according to age and region.

Early adulthood BMI was associated with all-cause mortality and cardiovascular disease mortality with a U-shaped relationship, which reached the lowest risk around 21·5 kg/m^2^ (appendix p 28). Compared with participants who had a typical early adulthood BMI (20·5–22·4 kg/m^2^), there was a higher risk of all-cause mortality and cardiovascular disease mortality for those who had an early adulthood BMI of less than 18·5 kg/m^2^ (all-cause HR 1·16 [95% CI 1·10–1·23]; cardiovascular disease HR 1·10 [95% CI 0·98–1·23]) or those with an early adulthood BMI of 30 kg/m^2^ or more (all-cause 1·29 [1·12–1·48]; cardiovascular 1·63 [1·27–2·09]; appendix p 8).

Sensitivity analyses supported the robustness of our main findings. Details of these results have been shown in appendix (pp 9–14).

In subgroup analysis by weight change, we found that the association between early adulthood BMI and later cardiovascular diseases robustly existed across different weight change groups (appendix pp 15–16). Considering the non-monotonic regularity of weight change, we further repeated the analysis in subgroups by age at baseline. Similar monotonic positive associations between early adulthood BMI and later health outcomes existed across different age groups. Additionally, our results showed that the associations were generally stronger among adults younger than 45 years after adjusting for weight change (appendix pp 17–18).

**Figure 2:**
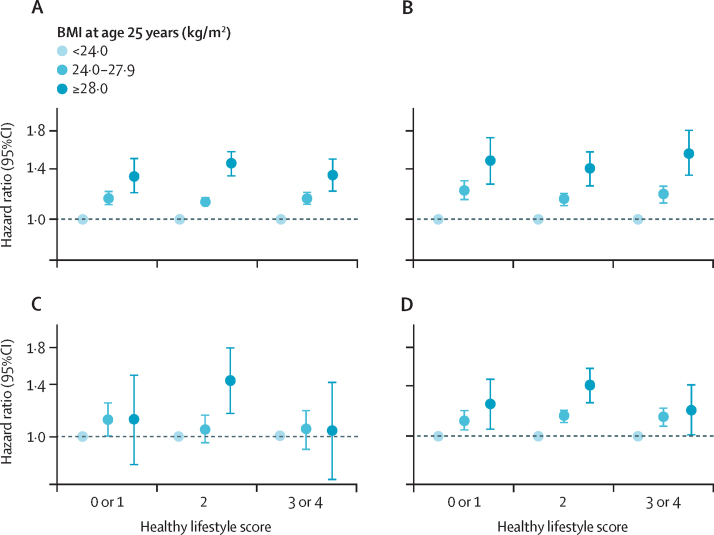
Association of early adulthood BMI with incident cardiovascular diseases by healthy lifestyle score Graphs show associations of early adulthood BMI with cardiovascular diseases generally (A), with ischaemic heart disease (B), with haemorrhagic stroke (C), and with ischaemic stroke (D). The analyses were adjusted for sex, education, marital status, hypertension status, family history, and weight change. Healthy lifestyle scores were merged to enhance the statistical power due to low numbers in the population.

Results showed that a one-point HLF score increase was associated with an approximately 20% lower risk of all-cause mortality (HR 0·80; 95% CI 0·79–0·82) and cardiovascular disease mortality (0·82; 0·79–0·84) and 12% lower risk of cardiovascular disease morbidity (0·88; 0·87–0·89; appendix p 19). However, the association of early adulthood BMI with cardiovascular diseases changed minimally in multivariable-adjusted models when we further adjusted for lifestyle factors in the model (table 2). Subgroup analysis showed that higher early adulthood BMI was associated with elevated risks of morbidity and mortality from cardiovascular diseases across various HLF score groups, and the hazard ratios were generally similar in magnitude (figure 2, appendix p 20). No significant multiplicative interaction was found between midlife HLFs and early adulthood BMI on cardiovascular disease outcomes after Bonferroni correction (appendix pp 22–25). The additive interaction between early adulthood BMI and midlife HLF score was also small, with the relative excess risks due to interaction ranging from –0·013 to 0·019 (appendix pp 26, 29).

## Discussion

This large and long-term cohort study increased understanding about early adulthood adiposity in the Chinese population. We found a monotonic positive association between higher early adulthood BMI and increased risks of incident cardiovascular diseases, ischaemic heart disease, haemorrhagic stroke, and ischaemic stroke, largely independent of subsequent weight change. Additionally, we found no significant interaction between early adulthood BMI and midlife HLFs on cardiovascular disease outcomes.

Several large prospective studies, primarily involving US and European populations, have investigated the association of early adulthood BMI with cardiovascular diseases; most of them showed positive associations.^5^, ^11^, ^20^ However, information from Asian populations is considerably scarce. Only two prospective cohort studies were done in east Asian countries, which had short follow-up times and unsatisfactory assessment of health outcomes (ie, self-reported cardiovascular disease without onset time as the study outcome).^10^, ^21^ In the current analyses, despite differences in fat distribution, lifestyle, and cultural background between people of Asian and European origin, we found a pattern of monotonic positive associations of early-life adiposity with cardiovascular diseases that was largely in agreement with previous findings from European and US studies. This result suggests that these associations are not unique to European and US populations but extend to Asian populations. For cardiovascular disease subtypes, our findings indicated that the association was slightly more prominent for ischaemic heart disease than stroke, which is comparable with a study of Swedish males, although our study had a smaller magnitude of association.^22^

The association between early adulthood BMI and stroke subtypes, particularly haemorrhagic stroke, still needs to be explored due to its relative rarity in other regions. A previous systematic review found that the pooled adjusted relative risk of ischaemic stroke was 1·40 (95% CI 1·24–1·58) for overweight and 1·78 (1·003–3·16) for obesity, whereas no significant association was found for haemorrhagic stroke with a wide confidence interval (pooled relative risk was 1·25 [95% CI 0·83–1·90] for overweight and 1·80 [0·97–3·35] for obesity).^11^ Our study, which included 5978 haemorrhagic stroke cases, suggests that obesity in early adulthood is associated with an elevated risk of haemorrhagic stroke in later life, with a similar strength of association to the risk of ischaemic stroke.

These associations can be attributed to several factors. First, redistribution of body fat from the extremities towards the trunk occurs during adolescence and early adulthood, leading to a predominance of central obesity.^23^ Second, early-life adiposity could lead to early onset and accumulation of vascular and metabolic risk factors, such as dyslipidaemia and hypertension, which could further contribute to the development of cardiovascular diseases along with excess bodyweight.^24^

We were also interested in the health consequences of having underweight in early adulthood. Our results of monotonic positive associations suggested people with underweight have a slightly reduced risk of ischaemic cardiovascular disease in later life. However, this finding is inconsistent with results from US and European populations, where most studies found similar risks between the groups with underweight and typical weights.^5^, ^22^ Conversely, two studies, one undertaken in a Chinese population and the other on Japanese American men, found a reduced risk for cardiovascular diseases with underweight.^10^, ^25^ The reason for this inconsistency is unclear, although the propensity for central adiposity in Asian populations might partly explain the discrepancy. Further studies are needed to substantiate differences between ethnicities.

In line with previous studies, our results showed that the associations between early adulthood BMI and cardiovascular diseases were largely independent of subsequent weight changes.^10^, ^26^ Our results also showed that these associations robustly existed across different age groups and were generally more substantial in the younger group. One possible explanation is that older individuals can have more weight fluctuation for a longer duration since early adulthood, which could dilute the association. Additionally, as higher early adulthood BMI is related to earlier onset of cardiovascular diseases, left censoring might result in an underestimation of the association, especially for individuals with older age during the baseline survey.

Previous studies did not give enough attention to the role of midlife HLFs in these associations.^5^, ^10^ In our study, despite the risk of multiple testing and small groups for subgroup analysis, we found that the effects of HLFs on modifying the associations between early-life adiposity and cardiovascular diseases were modest and that a substantial part of the increased mortality and morbidity might not be reversible in later years.

Some limitations of this study should be acknowledged. First, our study only included weight data for two timepoints and we could not assess dynamic weight changes and cumulative obesity exposure. The early weight information at 25 years of age relied on self-report in middle age, which could be subject to recall bias. Although the intraclass correlation coefficient was 0·81 in our data, and previous validation studies have also supported good validity of recalled early-life weight,^27^ some misclassifications are inevitable. However, given that weight data were obtained before the study outcomes, the misclassification would probably be non-differential, leading to an underestimation of the true association. In addition, the early adulthood BMI in our cohorts was similar to that of the general Chinese population during the corresponding period (mean 21·5 [SD 2·5] kg/m^2^ for adults aged 20–45 years in 1989).^28^ Second, some early adulthood lifestyle factors correlated with early BMI were not measured. Nevertheless, previous studies found little effect of early lifestyle factors on later health outcomes.^29^, ^30^ Moreover, in the sensitivity analysis, we included smoking and drinking status at age 25 years as potential confounders in the regression model and found that the association remained unchanged. This result aligned with another study that adjusted for various early lifestyle factors.^31^ Despite these reassurances, our results should be interpreted cautiously and warrant further research with a long-term follow-up that includes measurements of BMI and lifestyle factors in early adulthood to strengthen the evidence base. Third, the hazard ratio was dependent on the duration of the follow-up, which should be extrapolated with caution.^32^ Fourth, given the observational nature of our study design, we cannot entirely rule out residual confounding due to other unmeasured variables (eg, childhood factors) and imprecisely measured factors (eg, self-reported lifestyle factors in middle adulthood), even though the accuracy was validated and acceptable.

Our results showed that the prevalence of overweight and obesity increased substantially from 18% in early life to 44% in midlife. Findings from this study have crucial implications for public health, underlining the importance of weight management in early life. We want to emphasise that early adulthood is an imperative and relatively new target for obesity prevention, when people often have frequent changes, such as in weight and lifestyle, and are more likely to form lifelong habits that influence their weight. However, adults in this stage, who have fewer medical problems and perceive themselves as healthy, could often be unaware of the potential long-term adverse consequences of obesity.^33^ Furthermore, previous studies have found that weight loss interventions were generally difficult to achieve and hard to sustain,^34^ suggesting that the promotion of preventing excess weight gain in early adulthood might be more achievable than the loss of weight in people with obesity in later life. Therefore, public health programmes backed by effective government policies and clear action plans are needed to empower young adults to manage their health and prevent complications related to obesity and overweight and support them in adopting a healthy lifestyle, such as adequate physical activity and a balanced diet.

### Contributors

### Data sharing

Details of how to access China Kadoorie Biobank data and details of the data release schedule are available from http://www.ckbiobank.org/site/Data+Access. Data are shared with bona fide researchers; for specific criteria see https://www.ckbiobank.org/data-access/data-2.

## Declaration of interests

We declare no competing interests.
