# Supplementary material for: Early adulthood BMI and cardiovascular disease: a prospective cohort study from the China Kadoorie Biobank
Source: Lancet Public Health. 2024 Jun 15;9(12):e1005–13. doi: 10.1016/S2468-2667(24)00043-4 (PMC11617502; doi:10.1016/S2468-2667(24)00043-4)
Supplement: Supplementary appendix [file mmc1.pdf]

THE LANCET
Public Health

Supplementary appendix

This appendix formed part of the original submission and has been peer reviewed. We post it as supplied by the authors.

Supplement to: Chen Y, Yu W, Lv J, et al. Early adulthood BMI and cardiovascular disease: a prospective cohort study from the China Kadoorie Biobank. *Lancet Public Health* 2024; published online June 14. https://doi.org/10.1016/S2468-2667(24)00043-4.

Supplementary Appendix

Contents

Members of the China Kadoorie Biobank (CKB) collaborative group:..1
Abbreviations..2
Assessments of lifestyle factors 2
Assessment of other covariates 3
Statistical analyses 4
References..4
Table 1. Distribution of BMI at early/middle adulthood life [N (%)]. 5
Table 2. Associations of early adulthood BMI (4 groups) with mortality and morbidity of cardiovascular diseases (CVDs) [HR (95%CI)]...6
Table 3. Associations of early adulthood BMI (8 groups) with all-cause mortality and mortality of cardiovascular diseases (CVDs) [HR (95%CI)]...8
Table 4. Sensitivity analysis of associations of early adulthood BMI with all-cause mortality and mortality of cardiovascular diseases (CVDs)...9
Table 5. Sensitivity analysis (early lifestyles) of associations of early adulthood BMI with all-cause mortality and mortality of cardiovascular diseases (CVDs). 13
Table 6. Subgroup analysis of the associations of early adulthood BMI and health outcomes (by weight change, kg). 15
Table 7. Subgroup analysis of the associations of early adulthood BMI and health outcomes (by age at baseline, years). 17
Table 8. Hazard ratios (95% confidence intervals) of mortality and morbidity of CVDs associated with each healthy lifestyle factor and the healthy lifestyle score...19
Table 9. Subgroup analysis of the association between early adulthood BMI and health outcomes (by midlife healthy lifestyle score).*. 20
Table 10. Subgroup analysis of the association between early adulthood BMI and health outcomes (by midlife smoking habits).*. 22
Table 11. Subgroup analysis of the association between early adulthood BMI and health outcomes (by midlife drinking habits).*. 23
Table 12. Subgroup analysis of the association between early adulthood BMI and health outcomes (by midlife physical activity habits).*...24
Table 13. Subgroup analysis of the association between early adulthood BMI and health outcomes (by midlife diet habits).*. 25
Table 14. Additive interaction between early adulthood BMI and lifestyle factors on health outcomes. 26
Figure 1. Flowchart for inclusion and exclusion of the study sample*...27
Figure 2. Association of early adulthood BMI with all-cause mortality and mortality of cardiovascular diseases (CVDs): restricted cubic spline (RCS). 28
Figure 3. Attributable proportions from the early adulthood BMI, lifestyle score, and their interaction for incident CVDs*. 29

Members of the China Kadoorie Biobank (CKB) collaborative group:

International Steering Committee: Junshi Chen, Zhengming Chen (PI), Robert Clarke, Rory Collins, Yu Guo, Liming Li (PI), Jun Lv, Richard Peto, Robin Walters. **International Co-ordinating Centre, Oxford:** Daniel Avery, Derrick Bennett, Ruth Boxall, Sue Burgess, Ka Hung Chan, Yumei Chang, Yiping Chen, Zhengming Chen, Johnathan Clarke; Robert Clarke, Huaidong Du, Ahmed Edris Mohamed, Zammy Fairhurst-Hunter, Hannah Fry, Mike Hill, Michael Holmes, Pek Kei Im, Andri Iona, Maria Kakkoura, Christiana Kartsonaki, Rene Kerosi, Kuang Lin, Mohsen Mazidi, Iona Millwood, Sam Morris, Qunhua Nie, Alfred Pozarickij, Paul Ryder, Saredo Said, Dan Schmidt, Paul Sherliker, Becky Stevens, Iain Turnbull, Robin Walters, Lin Wang, Neil Wright, Ling Yang, Xiaoming Yang, Pang Yao. **National Co-ordinating Centre, Beijing:** Yu Guo, Xiao Han, Can Hou, Jun Lv, Pei Pei, Chao Liu, Canqing Yu, Qingmei Xia.

Regional Co-ordinating Centres: **Qingdao** CDC: Zengchang Pang, Ruqin Gao, Shanpeng Li, Haiping Duan, Shaojie Wang, Yongmei Liu, Ranran Du, Liang Cheng, Xiaocao Tian, Hua Zhang, Yaoming Zhai, Feng Ning, Xiaohui Sun, Feifei Li. Licang CDC: Silu Lv, Junzheng Wang, Wei Hou, Xiaoyan Zheng, **Heilongjiang** Provincial CDC: Wei Sun, Shichun Yan, Xiaoming Cui. Nangang CDC: Chi Wang, Zhenyuan Wu, Lishun Zhai, Yanjie Li, Zhaoxi Pang, Shiwen Dong. **Hainan** Provincial CDC: Huiming Luo, Yan Chen, Xingren Wang, Dingwei Sun, Tingting Ou. Meilan CDC: Xiangyang Zheng, Zhendong Guo, Shukuan Wu, Yilei Li, Lihui Li. **Jiangsu** Provincial CDC: Ming Wu, Yonglin Zhou, Jinyi Zhou, Ran Tao, Jian Su, Xikang Fan. Suzhou CDC: Fang Liu, Jun Zhang, Yan Lu, Li Xing, Yujie Hua, Jianrong Jin, Jingchao Liu. **Guangxi** Provincial CDC: Ge Zhong, Xianming Liao, Wei Mao,Zhenzhen Lu. Liuzhou CDC: Lifang Zhou, Zhongshu Qin, Jian Lan, Tingping Zhu, Liuping Wei, Liyuan Zhou, Sisi Wang. **Sichuan** Provincial CDC: Xianping Wu, Ningmei Zhang, Xiaofang Chen, Xiaoyu Chang. Pengzhou CDC: Mingqiang Yuan, Xia Wu, Xiaofang Chen, Wei Jiang, Jiaqiu Liu, Qiang Sun. **Gansu** Provincial CDC: Faqing Chen, Hupeng He, Xiaolan Ren. Maiji CDC: Zhongyi Hui, Jianjun Feng, Weijie Hu, Peng Wen, Yalin Chen, Fei Wang , Xiaofang Zhang. **Henan** Provincial CDC: Yibin Hao, Zhiwei Han, Kai Kang, Shixian Feng, Huizi Tian. Huixian CDC: Xiaolin Li, Huarong Sun, Pan He, Chen Hu, Xukui Zhang. **Zhejiang** Provincial CDC: Min Yu, Ruying Hu, Hao Wang, Weiwei Gong, Meng Wang. Tongxiang CDC: Huanxu Zhang, Yuan Cao, Kaixu Xie, Lingli Chen, Xiaomei Tu. **Hunan** Provincial CDC: Xiaojun Li, Li Yin, Huilin Liu, Donghui Jin. Liuyang CDC: Xin Xu, Jianwei Chen, Yuan Peng,

Chan Qu, Libo Zhang.

Abbreviations

BMI, body-mass index;

CVD, cardiovascular disease;

LF, lifestyle factor;

HS, haemorrhage stroke;

IHD, ischemic heart disease;

IS, ischemic stroke.

Assessments of lifestyle factors

Lifestyle factors included in this study were all obtained from the baseline questionnaire. For more on CKB questionnaires, see https://www.ckbiobank.org/.

1. Smoking/drinking (early adulthood)

Smoking/drinking status in early adulthood was assessed by the participant's recall of age starting habitual smoking/drinking in the questionnaire. By the age of 25 years old, the smoking/drinking status in early adulthood was categorized as "smoker/drinker" (starting age <25 years old) and "non-smoker/drinker" (starting age ≥ 25 years old).

2. Smoking (middle adulthood)

In the baseline questionnaire, questions about tobacco smoking included smoking status (never, former, or current smoker). Ever smokers were further asked about the frequency, type, and daily quantity of tobacco smoked. Additional inquiries were made to former smokers regarding the number of years since quitting and the reasons for their decision to quit. In this study, smoking behaviours were then categorized into five groups: never/occasional smokers, former smokers, 1-14 cigarettes/day, 15-24 cigarettes/day, and ≥25 cigarettes/day.

3. Drinking (middle adulthood)

In the baseline questionnaire, inquiry on alcohol intake included typical drinking frequency (never, occasionally, monthly, weekly, or daily). Drinkers with the frequency of at least once a week were subsequently asked about the type of alcoholic beverage drunk habitually, and the volume of alcohol drunk on a typical drinking day in the past 12 months. In this study, drinking behaviours were then classified into seven categories: non-weekly drinkers, former weekly drinkers, weekly but not daily drinkers, alcohol consumption less than 15 g/day, alcohol consumption of 15 to 29 g/day, alcohol consumption of 30 to 59 g/day, and alcohol consumption more than 60 g/day.

4. Physical activity

The methods we used to measure physical activity have been reported previously[1, 2] and were comparable with those utilized in previous studies, both in high-income countries[3] and Chinese demographics[4], though lack of a direct comparison with a reference methodology such as an accelerometer. Physical activity section in CKB baseline questionnaire included questions on the

intensity, frequency, and time spent on occupational tasks, commuting, household tasks, and leisure time activities. Metabolic equivalents of task (METs) of different types of activities were attained from the 2011 compendium of physical activities[5]. The total MET hours per day (MET-h/d) was calculated by sum of the product of frequency and duration of each physical activity.

5. Dietary habits

Dietary habits were assessed by validated food frequency questionnaire (FFQ), including habitual intakes of 12 conventional food groups during the past 12 months. The intake frequency was provided with 5 categories (daily consumption, 4-6 days/week, 1-3 days/week, several times a month, none/rarely). In this study, we consider the consumption of red meat, vegetables, fruits, and eggs by measuring frequencies.

The reproducibility and relative validity of baseline FFQ have been evaluated in previous study.[6] In brief, in 2015-2016, 432 participants from four CKB study sites were selected. The baseline FFQ's validity was analysed using 24-hour dietary recalls, and its reproducibility was confirmed by two FFQ administrations. Weighted kappa coefficients for all food groups, except fresh vegetables, exceeded 0.60 in both evaluations. Despite the low kappa for fresh vegetables, the percentage of correct classification was over 89% and the percentage of extreme classification was below 1% in both validity and reproducibility, indicating good validity and reproducibility of the FFQ.

6. Healthy lifestyle score

Above four modifiable lifestyle factors (smoking, drinking, physical activity, dietary habits) were dichotomized to construct a comprehensive healthy lifestyle score at midlife, based on previous studies[7, 8]. For smoking, we categorized current smokers and former smokers who quit because of illness into the unhealthy group. For alcohol drinking, those who consumed more than 30 g/day of pure alcohol or had ceased drinking were considered unhealthy. To avoid an overestimated risk for the reference group, we included former smokers and drinkers in the unhealthy group. For physical activity, individuals in the lower half of total physical activity for their sex- and age-specific group (<40, 40–50, and ≥50 years) were considered unhealthy. For dietary habits, based on Chinese dietary guidelines and previous findings in our population, unhealthy dietary habits were defined as non-daily consumption of vegetables, fruits, and eggs, and daily or less than weekly consumption of red meat. A healthy lifestyle score was simply derived based on the number of healthy lifestyle factors, ranging from 0 to 4, with higher scores indicating a healthier lifestyle.

Assessment of other covariates

Weight at baseline was measured to the nearest 0.1kg by trained staff using a body composition analyser (TANITA TBF-300GS; Tanita) in light clothing. Socio-demographic status and personal/family medical history were referred from the baseline questionnaire. Marital status was classified into four categories: Married, widowed, separated/divorced, and never married. Education was classified into six levels: illiterate, elementary, middle school, high school, junior college, college and above. Hypertension status was determined by the standard of (1) SBP≥140mmHg or DBP≥90mmHg

measured at baseline; (2) self-report of a prior diagnosis; (3) been taking anti-hypertensive agents. Family history of CMD was defined when participants had at least one parent or sibling suffering from stroke, heart attack, or diabetes.

Statistical analyses

1. Interaction between early adulthood BMI and midlife lifestyle factors

To explicitly investigate the potential effect modification of healthy lifestyles in middle adulthood, we conducted subgroup analysis by lifestyles and test the possible multiplicative and additive interactions between early adulthood BMI and midlife LFs (see supplementary for more detail). The likelihood test was used to examine multiplicative interaction by comparing regression models with and without cross-product terms. We also considered early adulthood BMI (per standard deviation increase, 2.5 kg/m^2) and numbers of healthy lifestyle factors (1 point per decrease) as continuous variables and tested for their additive interactions. Individual contributions from early adulthood BMI, lifestyle score, and the interaction between the two were assessed by relative excess risks due to interaction (RERI), attributable proportions of interaction (API), and their 95% CIs after centralization according to the previous study.[9, 10]

References

1. Du H, Bennett D, Li L, et al. Physical activity and sedentary leisure time and their associations with BMI, waist circumference, and percentage body fat in 0.5 million adults: the China Kadoorie Biobank study. Am J Clin Nutr. 2013;97(3):487-96.
2. Du H, Li L, Whitlock G, et al. Patterns and socio-demographic correlates of domain-specific physical activities and their associations with adiposity in the China Kadoorie Biobank study. Bmc Public Health. 2014;14:826.
3. Wareham NJ, Jakes RW, Rennie KL, Mitchell J, Hennings S, Day NE. Validity and repeatability of the EPIC-Norfolk Physical Activity Questionnaire. Int J Epidemiol. 2002;31(1):168-74.
4. Matthews CE, Shu XO, Yang G, et al. Reproducibility and validity of the Shanghai Women's Health Study physical activity questionnaire. Am J Epidemiol. 2003;158(11):1114-22.
5. Ainsworth BE, Haskell WL, Herrmann SD, et al. 2011 Compendium of Physical Activities: a second update of codes and MET values. Med Sci Sports Exerc. 2011;43(8):1575-81.
6. Zhu N, Yu C, Guo Y, et al. Adherence to a healthy lifestyle and all-cause and cause-specific mortality in Chinese adults: a 10-year prospective study of 0.5 million people. Int J Behav Nutr Phys Act. 2019;16(1):98.
7. Sun Q, Yu D, Fan J, et al. Healthy lifestyle and life expectancy at age 30 years in the Chinese population: an observational study. Lancet Public Health. 2022;7(12):e994-e1004.
8. Han Y, Hu Y, Yu C, et al. Lifestyle, cardiometabolic disease, and multimorbidity in a prospective Chinese study. Eur Heart J. 2021;42(34):3374-84.
9. VanderWeele TJ, Tchetgen Tchetgen EJ. Attributing effects to interactions. Epidemiology. 2014;25(5):711-22.
10. Balli HO, Sorensen BE. Interaction effects in econometrics. Empir Econ. 2013;45(1):583-603.

Table 1. Distribution of BMI at early/middle adulthood life [N (%)].

BMI at early adulthood (kg/m^2)	BMI at middle adulthood (kg/m^2)			Total
	<24.0	24.0~27.9	≥28.0	
<24.0	178,633 (49.5)	94,698 (26.2)	22,815 (6.3)	296,146 (82.1)
24.0~27.9	22,308 (6.2)	24,724 (6.9)	12,269 (3.4)	59,301 (16.4)
≥28.0	1138 (0.3)	2147 (0.6)	2123 (0.6)	5408 (1.5)
Total	202,079 (56)	121,569 (33.7)	37,207 (10.3)	360,855

Table 2. Associations of early adulthood BMI (4 groups) with mortality and morbidity of cardiovascular diseases (CVDs) [HR (95%CI)].

	Early Adulthood BMI (kg/m^2)				P_{trend}
	<18.5	18.5~23.9	24.0~27.9	≥28.0	
**Morbidity of Any CVD**					
No of case	4,453	40,773	10,702	1,275	
Incidence density (/1000 person year)	14.40 (13.98,14.83)	13.38 (13.25,13.51)	16.16 (15.86,16.47)	22.04 (20.86,23.28)	
Model 1	1.05 (1.01, 1.08)	1	1.06 (1.04, 1.08)	1.22 (1.16, 1.29)	<0.0001
Model 2	0.98 (0.95, 1.01)	1	1.13 (1.11, 1.16)	1.39 (1.32, 1.48)	<0.0001
Model 3	0.97 (0.94, 1.01)	1	1.13 (1.11, 1.16)	1.39 (1.31, 1.47)	<0.0001
**Morbidity of IHD**					
No of case	2,454	21,098	5,501	665	
Incidence density (/1000 person year)	7.71 (7.41,8.02)	6.74 (6.65,6.83)	8.04 (7.83,8.26)	11.01 (10.21,11.88)	
Model 1	1.06 (1.02, 1.11)	1	1.06 (1.03, 1.10)	1.22 (1.13, 1.32)	0.0004
Model 2	0.97 (0.93, 1.02)	1	1.16 (1.13, 1.20)	1.45 (1.34, 1.57)	<0.0001
Model 3	0.97 (0.93, 1.01)	1	1.16 (1.13, 1.20)	1.45 (1.34, 1.57)	<0.0001
**Morbidity of HS**					
No of case	402	4,155	1,250	171	
Incidence density (/1000 person year)	1.23 (1.11,1.35)	1.29 (1.26,1.33)	1.78 (1.68,1.88)	2.73 (2.35,3.17)	
Model 1	1.06 (0.95, 1.17)	1	1.13 (1.06, 1.20)	1.39 (1.19, 1.62)	<0.0001
Model 2	1.11 (1.00, 1.24)	1	1.08 (1.01, 1.15)	1.26 (1.08, 1.48)	0.058
Model 3	1.10 (0.99, 1.22)	1	1.08 (1.01, 1.15)	1.27 (1.08, 1.49)	0.038
**Morbidity of IS**					
No of case	2,282	21,575	5,693	642	
Incidence density (/1000 person year)	7.16 (6.87,7.46)	6.9 (6.8,6.99)	8.34 (8.12,8.56)	10.65 (9.86,11.51)	
Model 1	1.02 (0.98, 1.07)	1	1.06 (1.03, 1.09)	1.14 (1.06, 1.24)	0.00026
Model 2	0.95 (0.91, 1.00)	1	1.13 (1.10, 1.17)	1.31 (1.21, 1.42)	<0.0001
Model 3	0.95 (0.91, 0.99)	1	1.13 (1.10, 1.17)	1.31 (1.21, 1.42)	<0.0001

	Early Adulthood BMI (kg/m^2)				P_{trend}
	<18.5	18.5~23.9	24.0~27.9	≥28.0	
**All-cause Mortality**					
No of case	1,559	17,487	5,024	684	
Mortality density (/1000 person year)	4.74 (4.51,4.99)	5.43 (5.35,5.51)	7.11 (6.92,7.31)	10.85 (10.07,11.7)	
Model 1	1.02 (0.96, 1.07)	1	1.09 (1.05, 1.12)	1.41 (1.31, 1.52)	<0.0001
Model 2	1.16 (1.10, 1.23)	1	0.96 (0.93, 0.99)	1.10 (1.01, 1.19)	0.0024
Model 3	1.14 (1.08, 1.20)	1	0.96 (0.93, 1.00)	1.10 (1.02, 1.19)	0.013
**Mortality of Any CVD**					
No of case	407	4,356	1,338	203	
Mortality density (/1000 person year)	1.24 (1.12,1.36)	1.35 (1.31,1.39)	1.89 (1.80,2.00)	3.22 (2.81,3.70)	
Model 1	1.04 (0.94, 1.15)	1	1.11 (1.04, 1.18)	1.49 (1.29, 1.71)	<0.0001
Model 2	1.11 (1.00, 1.23)	1	1.05 (0.98, 1.12)	1.33 (1.15, 1.54)	0.051
Model 3	1.08 (0.97, 1.20)	1	1.06 (0.99, 1.13)	1.33 (1.15, 1.54)	0.024

In Model 1, hazard ratios were adjusted for sex, education, marital status, hypertension status, and family history; Model 2 adjusted for weight change based on Model 1; In Model 3, four lifestyle factors (smoking, alcohol consumption, physical activity, and diet) were further included. The analyses were stratified according to age and region.

Table 3. Associations of early adulthood BMI (8 groups) with all-cause mortality and mortality of cardiovascular diseases (CVDs) [HR (95%CI)].

	Early Adulthood BMI (kg/m^2)								P_{trend}
	<18.5	18.5~20.4	20.5~22.4	22.5~23.9	24.0~25.9	26.0~27.9	28.0~29.9	≥30.0	
**All-cause Mortality**									
No. of case	1,559	4,669	7,690	5,128	3,643	1,381	459	225	
Mortality density (/1000 person year)	4.74 (4.51,4.99)	4.62 (4.49,4.75)	5.41 (5.29,5.54)	6.50 (6.33,6.68)	6.88 (6.66,7.10)	7.82 (7.42,8.24)	9.80 (8.95,10.74)	13.90 (12.2,15.84)	
Model 1	1.03 (0.98, 1.09)	0.97 (0.94, 1.01)	1	1.09 (1.05, 1.13)	1.09 (1.05, 1.14)	1.15 (1.08, 1.21)	1.32 (1.20, 1.45)	1.77 (1.55, 2.02)	<0.0001
Model 2	1.18 (1.12, 1.25)	1.04 (0.99, 1.07)	1	1.03 (0.99, 1.07)	0.98 (0.94, 1.02)	0.97 (0.91, 1.03)	1.05 (0.96, 1.16)	1.29 (1.12, 1.48)	0.013
Model 3	1.16 (1.10, 1.23)	1.03 (0.99, 1.07)	1	1.04 (1.00, 1.07)	0.99 (0.95, 1.03)	0.98 (0.92, 1.04)	1.06 (0.96, 1.17)	1.29 (1.12, 1.48)	0.079
**Mortality of Any CVD**									
No. of case	407	1,163	1,894	1,299	965	373	135	68	
Mortality density (/1000 person year)	1.24 (1.12,1.36)	1.15 (1.09,1.22)	1.33 (1.27,1.40)	1.65 (1.56,1.74)	1.82 (1.71,1.94)	2.11 (1.91,2.34)	2.88 (2.44,3.41)	4.20 (3.31,5.33)	
Model 1	1.06 (0.96, 1.19)	0.98 (0.91, 1.06)	1	1.11 (1.03, 1.19)	1.14 (1.05, 1.23)	1.16 (1.03, 1.29)	1.40 (1.18, 1.67)	1.85 (1.45, 2.36)	<0.0001
Model 2	1.13 (1.01, 1.26)	1.01 (0.94, 1.08)	1	1.08 (1.01, 1.17)	1.09 (1.00, 1.18)	1.08 (0.96, 1.21)	1.28 (1.07, 1.53)	1.63 (1.27, 2.09)	0.0079
Model 3	1.10 (0.98, 1.23)	1.00 (0.93, 1.07)	1	1.09 (1.01, 1.17)	1.09 (1.01, 1.18)	1.09 (0.97, 1.22)	1.29 (1.07, 1.54)	1.63 (1.27, 2.09)	0.0020

In Model 1, hazard ratios were adjusted for sex, education, marital status, hypertension status, and family history; Model 2 adjusted for weight change based on Model 1; In Model 3, four lifestyle factors (smoking, alcohol consumption, physical activity, and diet) were further included. The analyses were stratified according to age and region.

Table 4. Sensitivity analysis of associations of early adulthood BMI with all-cause mortality and mortality of cardiovascular diseases (CVDs).

	Early Adulthood BMI (kg/m^2)							
	<18.5	18.5~20.4	20.5~22.4	22.5~23.9	24.0~25.9	26.0~27.9	28.0~29.9	≥30.0
**All-cause Mortality**								
Model 3	1.16 (1.10, 1.23)	1.03 (0.99, 1.07)	1	1.04 (1.00, 1.07)	0.99 (0.95, 1.03)	0.98 (0.92, 1.04)	1.06 (0.96, 1.17)	1.29 (1.12, 1.48)
In never/occasional smoker	1.11 (1.03, 1.20)	1.01 (0.96, 1.07)	1	1.04 (0.99, 1.10)	0.98 (0.92, 1.04)	0.97 (0.89, 1.06)	1.18 (1.03, 1.34)	1.46 (1.22, 1.76)
In females	1.11 (1.02, 1.20)	0.99 (0.94, 1.06)	1	0.99 (0.93, 1.06)	0.96 (0.89, 1.02)	0.97 (0.88, 1.06)	1.12 (0.98, 1.28)	1.33 (1.11, 1.60)
In males	1.18 (1.09, 1.28)	1.05 (1.00, 1.10)	1	1.06 (1.02, 1.11)	1.01 (0.96, 1.06)	0.98 (0.90, 1.06)	0.96 (0.83, 1.10)	1.16 (0.94, 1.43)
Exclude participants with follow-up < 2 yrs	1.15 (1.09, 1.22)	1.04 (0.99, 1.08)	1	1.04 (0.99, 1.08)	0.99 (0.95, 1.03)	0.99 (0.93, 1.06)	1.06 (0.96, 1.17)	1.33 (1.15, 1.53)
Exclude participants whose weight changed>2.5kg within 2 yrs	1.17 (1.10, 1.25)	1.04 (1.00, 1.09)	1	1.03 (0.99, 1.07)	0.99 (0.94, 1.04)	0.96 (0.89, 1.03)	1.05 (0.94, 1.18)	1.29 (1.08, 1.53)
Not adjust for hypertension	1.10 (1.04, 1.16)	1.01 (0.97, 1.04)	1	1.05 (1.02, 1.09)	1.02 (0.98, 1.07)	1.04 (0.98, 1.11)	1.16 (1.06, 1.28)	1.48 (1.29, 1.69)
Simultaneous adjustment for individual lifestyle factor score	1.17 (1.11, 1.24)	1.03 (1.00, 1.07)	1	1.04 (1.00, 1.07)	0.98 (0.94, 1.02)	0.97 (0.91, 1.03)	1.05 (0.95, 1.16)	1.28 (1.12, 1.47)
Additional adjustment for income level	1.17 (1.10, 1.23)	1.03 (0.99, 1.07)	1	1.04 (1.00, 1.07)	0.99 (0.95, 1.03)	0.97 (0.92, 1.03)	1.06 (0.96, 1.17)	1.28 (1.12, 1.47)
Imputation for early weight	1.12 (1.06, 1.18)	1.01 (0.97, 1.04)	1	1.04 (1.01, 1.07)	0.99 (0.95, 1.02)	0.98 (0.93, 1.03)	1.03 (0.94, 1.12)	1.21 (1.06, 1.38)
**Mortality of any CVD**								
Model 3	1.10 (0.98, 1.23)	1.00 (0.93, 1.07)	1	1.09 (1.01, 1.17)	1.09 (1.01, 1.18)	1.09 (0.97, 1.22)	1.29 (1.07, 1.54)	1.63 (1.27, 2.09)
In never/occasional smoker	1.11 (0.95, 1.29)	1.01 (0.90, 1.12)	1	1.13 (1.01, 1.25)	1.09 (0.97, 1.23)	1.18 (1.01, 1.39)	1.37 (1.08, 1.75)	1.58 (1.11, 2.24)
In females	1.10 (0.94, 1.30)	1.01 (0.89, 1.15)	1	1.12 (0.99, 1.28)	1.12 (0.97, 1.28)	1.21 (1.02, 1.44)	1.53 (1.20, 1.95)	1.76 (1.27, 2.45)
In males	1.11 (0.95, 1.30)	1.00 (0.91, 1.10)	1	1.08 (0.99, 1.18)	1.08 (0.98, 1.20)	0.99 (0.84, 1.16)	0.99 (0.74, 1.31)	1.33 (0.90, 1.98)
Exclude participants with follow-up < 2 yrs	1.11 (0.99, 1.25)	1.00 (0.93, 1.08)	1	1.12 (1.04, 1.21)	1.09 (1.00, 1.18)	1.09 (0.96, 1.23)	1.27 (1.05, 1.54)	1.57 (1.20, 2.05)
Exclude participants whose weight changed>2.5kg within 2 yrs	1.17 (1.03, 1.32)	1.02 (0.94, 1.11)	1	1.10 (1.01, 1.19)	1.13 (1.03, 1.24)	1.09 (0.95, 1.25)	1.20 (0.97, 1.50)	1.89 (1.40, 2.53)
Not adjust for hypertension	0.96 (0.86, 1.08)	0.94 (0.88, 1.02)	1	1.13 (1.06, 1.22)	1.19 (1.10, 1.29)	1.28 (1.14, 1.43)	1.61 (1.34, 1.92)	2.25 (1.76, 2.89)
Simultaneous adjustment for individual lifestyle factor score	1.12 (1.00, 1.25)	1.01 (0.93, 1.08)	1	1.09 (1.01, 1.17)	1.09 (1.01, 1.18)	1.08 (0.96, 1.21)	1.27 (1.06, 1.53)	1.63 (1.27, 2.09)

	Early Adulthood BMI (kg/m²)							
	<18.5	18.5~20.4	20.5~22.4	22.5~23.9	24.0~25.9	26.0~27.9	28.0~29.9	≥30.0
Additional adjustment for income level	1.10 (0.98, 1.22)	1.00 (0.93, 1.07)	1	1.09 (1.02, 1.17)	1.09 (1.01, 1.18)	1.09 (0.97, 1.23)	1.29 (1.08, 1.55)	1.63 (1.27, 2.09)
Competing risks regression	1.13 (1.00,1.26)	1.01 (0.93,1.09)	1	1.05 (0.97,1.13)	1.05 (0.95,1.15)	1.01 (0.87,1.15)	1.18 (0.95,1.41)	1.45 (1.05,1.85)
Imputation for early weight	1.02 (0.92, 1.14)	0.95 (0.89, 1.01)	1	1.10 (1.04, 1.16)	1.08 (1.02, 1.16)	1.11 (1.00, 1.22)	1.23 (1.04, 1.45)	1.43 (1.12, 1.83)
**Morbidity of any CVD**								
Model 3	0.97 (0.94, 1.00)	0.97 (0.95, 0.99)	1	1.04 (1.02, 1.07)	1.12 (1.09, 1.15)	1.19 (1.14, 1.24)	1.34 (1.25, 1.44)	1.58 (1.42, 1.75)
In never/occasional smoker	0.94 (0.90, 0.98)	0.96 (0.93, 0.98)	1	1.03 (1.00, 1.07)	1.11 (1.07, 1.14)	1.15 (1.10, 1.21)	1.31 (1.21, 1.43)	1.53 (1.34, 1.75)
In females	0.95 (0.91, 0.99)	0.96 (0.93, 0.99)	1	1.03 (0.99, 1.06)	1.11 (1.07, 1.15)	1.16 (1.10, 1.22)	1.32 (1.21, 1.44)	1.54 (1.35, 1.76)
In males	0.97 (0.91, 1.03)	0.97 (0.93, 1.00)	1	1.06 (1.02, 1.09)	1.14 (1.10, 1.19)	1.27 (1.19, 1.36)	1.40 (1.26, 1.57)	1.69 (1.43, 2.00)
Exclude participants with follow-up < 2 yrs	0.97 (0.93, 1.00)	0.97 (0.94, 0.99)	1	1.04 (1.02, 1.07)	1.13 (1.10, 1.16)	1.21 (1.16, 1.26)	1.35 (1.26, 1.45)	1.59 (1.43, 1.78)
Exclude participants whose weight changed>2.5kg within 2 yrs	0.97 (0.93, 1.00)	0.97 (0.94, 0.99)	1	1.03 (1.00, 1.06)	1.11 (1.08, 1.15)	1.19 (1.13, 1.24)	1.30 (1.20, 1.41)	1.54 (1.35, 1.75)
Not adjust for hypertension	0.90 (0.87, 0.93)	0.94 (0.92, 0.96)	1	1.07 (1.04, 1.09)	1.19 (1.15, 1.22)	1.31 (1.26, 1.37)	1.54 (1.44, 1.65)	1.92 (1.73, 2.13)
Simultaneous adjustment for individual lifestyle factor score	0.97 (0.94, 1.00)	0.97 (0.95, 0.99)	1	1.04 (1.02, 1.07)	1.12 (1.09, 1.16)	1.19 (1.15, 1.24)	1.35 (1.26, 1.44)	1.59 (1.43, 1.76)
Additional adjustment for income level	0.97 (0.94, 1.00)	0.97 (0.95, 0.99)	1	1.04 (1.02, 1.07)	1.12 (1.09, 1.15)	1.19 (1.14, 1.24)	1.34 (1.25, 1.43)	1.58 (1.42, 1.75)
Competing risks regression	0.98 (0.95,1.02)	0.98 (0.96,1.00)	1	1.03 (1.00,1.05)	1.11 (1.08,1.14)	1.18 (1.12,1.24)	1.31 (1.18,1.43)	1.49 (1.26,1.73)
Imputation for early weight	0.96 (0.93, 1.00)	0.96 (0.94, 0.99)	1	1.05 (1.03, 1.07)	1.11 (1.09, 1.14)	1.18 (1.14, 1.23)	1.32 (1.23, 1.41)	1.54 (1.39, 1.71)
**Morbidity of IHD**								
Model 3	0.96 (0.92, 1.01)	0.96 (0.93, 0.99)	1	1.05 (1.02, 1.09)	1.15 (1.10, 1.19)	1.24 (1.17, 1.31)	1.40 (1.27, 1.53)	1.68 (1.45, 1.93)
In never/occasional smoker	0.94 (0.89, 0.99)	0.97 (0.93, 1.01)	1	1.06 (1.01, 1.11)	1.14 (1.08, 1.19)	1.21 (1.13, 1.29)	1.40 (1.25, 1.58)	1.67 (1.39, 2.00)
In females	0.94 (0.89, 0.99)	0.96 (0.92, 1.01)	1	1.04 (0.99, 1.09)	1.15 (1.09, 1.20)	1.21 (1.13, 1.30)	1.43 (1.27, 1.61)	1.69 (1.41, 2.02)
In males	0.99 (0.91, 1.07)	0.95 (0.91, 1.00)	1	1.06 (1.01, 1.11)	1.15 (1.09, 1.22)	1.31 (1.20, 1.44)	1.34 (1.14, 1.58)	1.69 (1.32, 2.16)
Exclude participants with follow-up < 2 yrs	0.95 (0.91, 1.00)	0.97 (0.93, 0.99)	1	1.05 (1.02, 1.09)	1.15 (1.10, 1.20)	1.25 (1.18, 1.32)	1.41 (1.27, 1.55)	1.71 (1.47, 1.99)

	Early Adulthood BMI (kg/m²)							
	<18.5	18.5~20.4	20.5~22.4	22.5~23.9	24.0~25.9	26.0~27.9	28.0~29.9	≥30.0
Exclude participants whose weight changed>2.5kg within 2 yrs	0.95 (0.90, 1.00)	0.97 (0.93, 1.01)	1	1.04 (1.00, 1.08)	1.14 (1.09, 1.19)	1.21 (1.14, 1.30)	1.40 (1.25, 1.57)	1.56 (1.30, 1.88)
Not adjust for hypertension	0.92 (0.88, 0.96)	0.94 (0.91, 0.97)	1	1.07 (1.03, 1.11)	1.19 (1.15, 1.24)	1.32 (1.25, 1.40)	1.53 (1.39, 1.68)	1.92 (1.66, 2.21)
Simultaneous adjustment for individual lifestyle factor score	0.96 (0.92, 1.01)	0.96 (0.93, 0.99)	1	1.05 (1.02, 1.09)	1.15 (1.11, 1.19)	1.24 (1.17, 1.31)	1.40 (1.28, 1.54)	1.68 (1.46, 1.94)
Additional adjustment for income level	0.96 (0.92, 1.01)	0.96 (0.93, 1.00)	1	1.05 (1.02, 1.09)	1.15 (1.11, 1.19)	1.24 (1.17, 1.31)	1.40 (1.28, 1.54)	1.69 (1.46, 1.95)
Competing risks regression	0.97 (0.93,1.02)	0.98 (0.94,1.01)	1	1.03 (0.99,1.07)	1.13 (1.09,1.18)	1.21 (1.14,1.28)	1.29 (1.16,1.41)	1.43 (1.21,1.66)
Imputation for early weight	0.96 (0.92, 1.00)	0.96 (0.93, 0.99)	1	1.05 (1.02, 1.08)	1.13 (1.09, 1.17)	1.22 (1.15, 1.28)	1.37 (1.25, 1.51)	1.65 (1.43, 1.90)
**Morbidity of HS**								
Model 3	1.10 (0.98, 1.23)	0.97 (0.90, 1.05)	1	1.03 (0.96, 1.11)	1.10 (1.01, 1.19)	1.06 (0.94, 1.20)	1.16 (0.95, 1.41)	1.61 (1.24, 2.09)
In never/occasional smoker	1.04 (0.90, 1.20)	0.96 (0.86, 1.06)	1	0.98 (0.88, 1.09)	1.03 (0.92, 1.16)	1.03 (0.88, 1.22)	1.11 (0.86, 1.43)	1.39 (0.96, 1.99)
In females	1.06 (0.91, 1.23)	0.89 (0.79, 1.00)	1	0.94 (0.83, 1.05)	1.00 (0.88, 1.13)	1.00 (0.85, 1.19)	1.08 (0.83, 1.40)	1.40 (0.99, 1.98)
In males	1.13 (0.96, 1.33)	1.02 (0.93, 1.13)	1	1.10 (1.00, 1.21)	1.16 (1.04, 1.29)	1.07 (0.90, 1.27)	1.16 (0.86, 1.57)	1.71 (1.14, 2.57)
Exclude participants with follow-up < 2 yrs	1.13 (1.00, 1.27)	0.99 (0.91, 1.07)	1	1.07 (0.99, 1.16)	1.10 (1.01, 1.20)	1.07 (0.94, 1.21)	1.16 (0.94, 1.43)	1.60 (1.21, 2.12)
Exclude participants whose weight changed>2.5kg within 2 yrs	1.11 (0.97, 1.26)	0.96 (0.88, 1.05)	1	1.02 (0.94, 1.11)	1.11 (1.01, 1.22)	1.03 (0.90, 1.19)	1.03 (0.80, 1.32)	1.70 (1.24, 2.33)
Not adjust for hypertension	0.92 (0.82, 1.03)	0.90 (0.83, 0.97)	1	1.10 (1.02, 1.18)	1.25 (1.15, 1.35)	1.32 (1.17, 1.49)	1.56 (1.28, 1.90)	2.50 (1.92, 3.24)
Simultaneous adjustment for individual lifestyle factor score	1.11 (0.99, 1.24)	0.98 (0.90, 1.05)	1	1.03 (0.96, 1.11)	1.09 (1.01, 1.19)	1.05 (0.93, 1.19)	1.14 (0.94, 1.39)	1.60 (1.23, 2.08)
Additional adjustment for income level	1.10 (0.99, 1.23)	0.97 (0.90, 1.05)	1	1.03 (0.96, 1.11)	1.10 (1.01, 1.19)	1.06 (0.94, 1.19)	1.15 (0.94, 1.40)	1.60 (1.23, 2.08)
Competing risks regression	0.97 (0.85,1.09)	0.94 (0.86,1.01)	1	0.99 (0.91,1.07)	1.05 (0.96,1.14)	0.98 (0.85,1.10)	0.94 (0.72,1.16)	1.34 (0.93,1.76)
Imputation for early weight	1.09 (0.98, 1.21)	0.96 (0.89, 1.02)	1	1.07 (1.01, 1.14)	1.10 (1.02, 1.17)	1.09 (0.98, 1.21)	1.17 (0.97, 1.40)	1.59 (1.24, 2.06)
**Morbidity of IS**								
Model 3	0.94 (0.89, 0.99)	0.96 (0.93, 1.00)	1	1.03 (0.99, 1.08)	1.11 (1.06, 1.16)	1.18 (1.10, 1.27)	1.24 (1.10, 1.40)	1.44 (1.19, 1.73)
In never/occasional smoker	0.94 (0.89, 0.99)	0.97 (0.93, 1.00)	1	1.03 (0.99, 1.08)	1.11 (1.06, 1.16)	1.18 (1.10, 1.27)	1.24 (1.10, 1.40)	1.44 (1.19, 1.73)

	Early Adulthood BMI (kg/m²)							
	<18.5	18.5~20.4	20.5~22.4	22.5~23.9	24.0~25.9	26.0~27.9	28.0~29.9	≥30.0
In females	0.95 (0.89, 1.01)	0.97 (0.93, 1.02)	1	1.03 (0.98, 1.08)	1.11 (1.05, 1.16)	1.18 (1.10, 1.27)	1.20 (1.06, 1.36)	1.42 (1.17, 1.72)
In males	0.93 (0.86, 1.01)	0.98 (0.94, 1.03)	1	1.06 (1.01, 1.11)	1.14 (1.08, 1.21)	1.29 (1.19, 1.41)	1.41 (1.22, 1.64)	1.54 (1.22, 1.95)
Exclude participants with follow-up < 2 yrs	0.95 (0.91, 1.00)	0.98 (0.94, 1.01)	1	1.04 (1.01, 1.08)	1.13 (1.09, 1.17)	1.24 (1.17, 1.31)	1.29 (1.17, 1.42)	1.46 (1.26, 1.71)
Exclude participants whose weight changed>2.5kg within 2 yrs	0.97 (0.91, 1.02)	0.97 (0.94, 1.01)	1	1.04 (0.99, 1.08)	1.12 (1.07, 1.17)	1.22 (1.15, 1.30)	1.24 (1.11, 1.40)	1.44 (1.19, 1.73)
Not adjust for hypertension	0.88 (0.84, 0.92)	0.95 (0.92, 0.98)	1	1.08 (1.04, 1.11)	1.19 (1.15, 1.24)	1.35 (1.28, 1.43)	1.49 (1.35, 1.63)	1.82 (1.57, 2.11)
Simultaneous adjustment for individual lifestyle factor score	0.95 (0.91, 1.00)	0.98 (0.95, 1.01)	1	1.04 (1.01, 1.08)	1.12 (1.08, 1.16)	1.22 (1.15, 1.29)	1.28 (1.17, 1.41)	1.47 (1.27, 1.70)
Additional adjustment for income level	0.95 (0.91, 1.00)	0.98 (0.95, 1.01)	1	1.04 (1.01, 1.08)	1.12 (1.08, 1.16)	1.22 (1.15, 1.29)	1.28 (1.17, 1.41)	1.47 (1.27, 1.70)
Competing risks regression	0.96 (0.91,1.00)	0.99 (0.96,1.02)	1	1.03 (0.99,1.06)	1.10 (1.06,1.14)	1.19 (1.12,1.25)	1.19 (1.07,1.31)	1.20 (1.00,1.39)
Imputation for early weight	0.94 (0.90, 0.99)	0.97 (0.94, 1.00)	1	1.05 (1.02, 1.08)	1.11 (1.08, 1.15)	1.20 (1.14, 1.27)	1.25 (1.14, 1.37)	1.43 (1.24, 1.66)

Table 5. Sensitivity analysis (early lifestyles) of associations of early adulthood BMI with all-cause mortality and mortality of cardiovascular diseases (CVDs).

	Early Adulthood BMI (kg/m^2)							
	<18.5	18.5~20.4	20.5~22.4	22.5~23.9	24.0~25.9	26.0~27.9	28.0~29.9	≥30.0
**All-cause Mortality**								
Model 1	1.03 (0.98, 1.09)	0.97 (0.94, 1.01)	1	1.09 (1.05, 1.13)	1.09 (1.05, 1.14)	1.15 (1.08, 1.21)	1.32 (1.20, 1.45)	1.77 (1.55, 2.02)
Model 2	1.03 (0.98, 1.09)	0.98 (0.94, 1.01)	1	1.09 (1.05, 1.13)	1.09 (1.05, 1.14)	1.14 (1.08, 1.21)	1.30 (1.19, 1.43)	1.71 (1.50, 1.96)
Model 3	1.18 (1.11, 1.25)	1.03 (1.00, 1.07)	1	1.03 (1.00, 1.07)	0.99 (0.95, 1.03)	0.97 (0.92, 1.03)	1.05 (0.96, 1.16)	1.27 (1.11, 1.46)
Model 4	1.16 (1.10, 1.23)	1.03 (0.99, 1.07)	1	1.04 (1.00, 1.07)	0.99 (0.95, 1.03)	0.98 (0.92, 1.04)	1.06 (0.96, 1.16)	1.28 (1.12, 1.47)
**CVD Mortality**								
Model 1	1.07 (0.96, 1.19)	0.98 (0.91, 1.06)	1	1.11 (1.03, 1.19)	1.13 (1.05, 1.23)	1.15 (1.03, 1.29)	1.39 (1.16, 1.65)	1.80 (1.41, 2.30)
Model 2	1.12 (1.00, 1.25)	1.00 (0.93, 1.08)	1	1.09 (1.01, 1.17)	1.09 (1.00, 1.18)	1.09 (0.97, 1.22)	1.29 (1.08, 1.55)	1.63 (1.27, 2.09)
Model 3	1.12 (1.00, 1.25)	1.00 (0.93, 1.08)	1	1.09 (1.01, 1.17)	1.09 (1.00, 1.18)	1.09 (0.97, 1.22)	1.29 (1.08, 1.55)	1.63 (1.27, 2.09)
Model 4	1.10 (0.98, 1.22)	1.00 (0.93, 1.07)	1	1.09 (1.02, 1.17)	1.09 (1.01, 1.18)	1.09 (0.97, 1.23)	1.29 (1.08, 1.55)	1.63 (1.27, 2.09)
**Morbidity of Any CVD**								
Model 1	1.05 (1.02, 1.09)	1.00 (0.98, 1.03)	1	1.01 (0.98, 1.03)	1.06 (1.03, 1.09)	1.08 (1.04, 1.13)	1.18 (1.11, 1.27)	1.32 (1.19, 1.46)
Model 2	0.97 (0.94, 1.01)	0.97 (0.95, 0.99)	1	1.04 (1.01, 1.07)	1.12 (1.09, 1.15)	1.19 (1.15, 1.24)	1.34 (1.26, 1.44)	1.59 (1.43, 1.76)
Model 3	0.97 (0.94, 1.01)	0.97 (0.95, 0.99)	1	1.04 (1.01, 1.07)	1.12 (1.09, 1.15)	1.19 (1.15, 1.24)	1.34 (1.26, 1.44)	1.59 (1.43, 1.76)
Model 4	0.97 (0.94, 1.00)	0.97 (0.95, 0.99)	1	1.04 (1.02, 1.07)	1.12 (1.09, 1.15)	1.19 (1.14, 1.24)	1.34 (1.25, 1.43)	1.58 (1.42, 1.75)
**Morbidity of IHD**								
Model 1	1.07 (1.02, 1.12)	1.01 (0.98, 1.04)	1	1.01 (0.97, 1.04)	1.06 (1.02, 1.10)	1.09 (1.04, 1.16)	1.18 (1.08, 1.30)	1.33 (1.16, 1.54)
Model 2	1.07 (1.02, 1.12)	1.01 (0.98, 1.04)	1	1.01 (0.97, 1.04)	1.06 (1.02, 1.10)	1.09 (1.03, 1.15)	1.18 (1.08, 1.30)	1.32 (1.14, 1.52)
Model 3	0.96 (0.92, 1.01)	0.96 (0.93, 1.00)	1	1.05 (1.02, 1.09)	1.15 (1.11, 1.20)	1.24 (1.17, 1.31)	1.41 (1.28, 1.55)	1.67 (1.45, 1.93)
Model 4	0.96 (0.92, 1.01)	0.96 (0.93, 1.00)	1	1.05 (1.02, 1.09)	1.15 (1.10, 1.19)	1.24 (1.17, 1.31)	1.40 (1.27, 1.53)	1.67 (1.45, 1.93)
**Morbidity of HS**								
Model 1	1.06 (0.95, 1.18)	0.95 (0.89, 1.03)	1	1.05 (0.98, 1.13)	1.14 (1.05, 1.23)	1.12 (0.99, 1.26)	1.24 (1.02, 1.50)	1.79 (1.39, 2.32)

	Early Adulthood BMI (kg/m^2)							
	<18.5	18.5~20.4	20.5~22.4	22.5~23.9	24.0~25.9	26.0~27.9	28.0~29.9	≥30.0
Model 2	1.06 (0.95, 1.18)	0.96 (0.89, 1.03)	1	1.05 (0.98, 1.13)	1.14 (1.05, 1.23)	1.11 (0.99, 1.25)	1.24 (1.02, 1.50)	1.78 (1.37, 2.30)
Model 3	1.11 (0.99, 1.24)	0.98 (0.90, 1.05)	1	1.03 (0.96, 1.11)	1.10 (1.01, 1.19)	1.05 (0.93, 1.19)	1.15 (0.94, 1.40)	1.60 (1.23, 2.08)
Model 4	1.10 (0.98, 1.23)	0.97 (0.90, 1.05)	1	1.03 (0.96, 1.11)	1.10 (1.01, 1.19)	1.06 (0.94, 1.20)	1.15 (0.95, 1.40)	1.61 (1.24, 2.09)
**Morbidity of IS**								
Model 1	1.03 (0.99, 1.08)	1.02 (0.99, 1.05)	1	1.01 (0.98, 1.04)	1.05 (1.01, 1.09)	1.11 (1.05, 1.17)	1.12 (1.02, 1.24)	1.23 (1.06, 1.42)
Model 2	1.03 (0.99, 1.08)	1.02 (0.99, 1.05)	1	1.01 (0.98, 1.04)	1.05 (1.01, 1.09)	1.10 (1.05, 1.16)	1.12 (1.02, 1.23)	1.22 (1.05, 1.41)
Model 3	0.95 (0.91, 1.00)	0.98 (0.95, 1.02)	1	1.04 (1.01, 1.08)	1.12 (1.08, 1.17)	1.22 (1.16, 1.29)	1.29 (1.17, 1.42)	1.47 (1.27, 1.70)
Model 4	0.95 (0.91, 1.00)	0.98 (0.95, 1.01)	1	1.05 (1.01, 1.08)	1.12 (1.08, 1.16)	1.22 (1.15, 1.29)	1.28 (1.16, 1.41)	1.46 (1.26, 1.70)

In Model 1, hazard ratios were adjusted for sex, education, marital status, hypertension status, and family history (same to the main analysis); Model 2 adjusted for smoking and drinking in early adulthood based on Model 1; Model 3 adjusted for weight change based on Model 2; In Model 4, four lifestyle factors in middle adulthood (smoking, alcohol consumption, physical activity, diet) were further included. The analyses were stratified according to age and region.

Table 6. Subgroup analysis of the associations of early adulthood BMI and health outcomes (by weight change, kg).

		Early adulthood BMI (kg/m^2)				$P_{interaction}$
		<18.5	18.5~23.9	24.0~27.9	≥28.0	
Morbidity of CVD						0.41
<-2.5	N. of case	82	5,725	4,310	864	
	HR (95%CI)	1.22 (0.98, 1.52)	1	1.10 (1.05, 1.15)	1.31 (1.21, 1.42)	
-2.5~2.5	N. of case	374	7,215	2,222	192	
	HR (95%CI)	0.96 (0.86, 1.06)	1	1.12 (1.07, 1.18)	1.27 (1.10, 1.47)	
≥2.5	N. of case	3,997	27,833	4,170	219	
	HR (95%CI)	0.96 (0.93, 0.99)	1	1.14 (1.10, 1.18)	1.31 (1.15, 1.50)	
Morbidity of IHD						0.11
<-2.5	N. of case	49	2,753	2,105	459	
	HR (95%CI)	1.43 (1.08, 1.90)	1	1.11 (1.05, 1.18)	1.44 (1.30, 1.59)	
-2.5~2.5	N. of case	194	3,485	1107	91	
	HR (95%CI)	0.97 (0.84, 1.12)	1	1.15 (1.07, 1.23)	1.19 (0.97, 1.47)	
≥2.5	N. of case	2,211	14,860	2,289	115	
	HR (95%CI)	1.01 (0.97, 1.06)	1	1.13 (1.08, 1.18)	1.22 (1.02, 1.47)	
Morbidity of HS						0.37
<-2.5	N. of case	6	844	626	131	
	HR (95%CI)	0.68 (0.30, 1.52)	1	1.06 (0.96, 1.18)	1.31 (1.08, 1.58)	
-2.5~2.5	N. of case	50	908	235	20	
	HR (95%CI)	1.07 (0.80, 1.43)	1	0.96 (0.83, 1.12)	1.04 (0.67, 1.63)	
≥2.5	N. of case	346	2,403	389	20	
	HR (95%CI)	1.12 (1.00, 1.26)	1	1.15 (1.03, 1.28)	1.17 (0.76, 1.83)	
Morbidity of IS						0.24
<-2.5	N. of case	37	2,834	2,242	415	
	HR (95%CI)	1.12 (0.81, 1.55)	1	1.14 (1.08, 1.20)	1.24 (1.11, 1.38)	
-2.5~2.5	N. of case	172	3,777	1230	104	
	HR (95%CI)	0.90 (0.77, 1.05)	1	1.14 (1.06, 1.21)	1.23 (1.01, 1.50)	
≥2.5	N. of case	2,073	14,964	2,221	123	
	HR (95%CI)	0.99 (0.94, 1.03)	1	1.09 (1.04, 1.14)	1.30 (1.09, 1.55)	
All-cause mortality						0.0006
<-2.5	N. of case	81	4,628	2,878	530	
	HR (95%CI)	1.57 (1.26, 1.96)	1	0.96 (0.91, 1.00)	1.14 (1.04, 1.25)	
-2.5~2.5	N. of case	245	3,890	922	77	
	HR (95%CI)	1.21 (1.06, 1.38)	1	0.94 (0.87, 1.01)	1.09 (0.86, 1.36)	
≥2.5	N. of case	1,233	8,969	1,224	77	
	HR (95%CI)	1.06 (0.99, 1.12)	1	1.04 (0.98, 1.11)	1.43 (1.14, 1.79)	
Mortality of CVD						0.15
<-2.5	N. of case	20	1,045	720	150	
	HR (95%CI)	1.84 (1.17, 2.87)	1	0.92 (0.83, 1.02)	0.97 (0.80, 1.18)	
-2.5~2.5	N. of case	58	955	263	24	
	HR (95%CI)	1.10 (0.84, 1.44)	1	1.06 (0.92, 1.22)	1.32 (0.88, 1.99)	
≥2.5	N. of case	329	2,356	355	29	

	Early adulthood BMI (kg/m^2)				$P_{interaction}$
	<18.5	18.5~23.9	24.0~27.9	≥28.0	
HR (95%CI)	1.02 (0.90, 1.15)	1	1.12 (1.00, 1.26)	1.89 (1.31, 2.73)	

HRs were stratified by age and study regions, and adjusted for sex, education, marital status, hypertension, family history, individual lifestyles and weight change.

Table 7. Subgroup analysis of the associations of early adulthood BMI and health outcomes (by age at baseline, years).

		Early adulthood BMI (kg/m^2)				$P_{interaction}$
		<18.5	18.5~23.9	24.0~27.9	≥28.0	
Morbidity of CVD						0.0002
<45	N. of case	781	6,116	1,233	171	
	HR (95%CI)	1.06 (0.99, 1.15)	1	1.23 (1.15, 1.30)	1.69 (1.45, 1.97)	
45~65	N. of case	3,005	28,412	7,354	767	
	HR (95%CI)	0.94 (0.91, 0.98)	1	1.10 (1.08, 1.14)	1.32 (1.23, 1.42)	
≥65	N. of case	667	6,245	2,115	337	
	HR (95%CI)	1.03 (0.94, 1.11)	1	1.12 (1.07, 1.19)	1.33 (1.18, 1.49)	
Morbidity of IHD						0.070
<45	N. of case	460	3,218	611	88	
	HR (95%CI)	1.10 (1.00, 1.22)	1	1.22 (1.11, 1.33)	1.75 (1.41, 2.17)	
45~65	N. of case	1,626	14,533	3,812	397	
	HR (95%CI)	0.93 (0.88, 0.98)	1	1.16 (1.12, 1.20)	1.41 (1.27, 1.56)	
≥65	N. of case	368	3,347	1,078	180	
	HR (95%CI)	1.00 (0.90, 1.12)	1	1.09 (1.01, 1.18)	1.32 (1.12, 1.54)	
Morbidity of HS						0.45
<45	N. of case	71	593	131	20	
	HR (95%CI)	1.27 (0.98, 1.63)	1	1.11 (0.92, 1.35)	1.49 (0.95, 2.35)	
45~65	N. of case	251	2,779	794	89	
	HR (95%CI)	1.05 (0.92, 1.21)	1	1.04 (0.96, 1.13)	1.15 (0.93, 1.43)	
≥65	N. of case	80	783	325	62	
	HR (95%CI)	1.18 (0.93, 1.49)	1	1.12 (0.97, 1.29)	1.34 (1.01, 1.77)	
Morbidity of IS						0.0071
<45	N. of case	311	2,701	573	78	
	HR (95%CI)	0.97 (0.86, 1.09)	1	1.26 (1.15, 1.39)	1.69 (1.34, 2.12)	
45~65	N. of case	1,610	15,370	3,942	393	
	HR (95%CI)	0.94 (0.89, 0.99)	1	1.09 (1.05, 1.13)	1.22 (1.10, 1.35)	
≥65	N. of case	361	3,504	1,178	171	
	HR (95%CI)	0.97 (0.87, 1.09)	1	1.17 (1.09, 1.25)	1.31 (1.12, 1.54)	
All-cause mortality						0.72
<45	N. of case	226	2,129	380	39	
	HR (95%CI)	1.17 (1.02, 1.35)	1	1.00 (0.90, 1.12)	1.01 (0.73, 1.39)	
45~65	N. of case	941	11,078	3,109	372	
	HR (95%CI)	1.12 (1.05, 1.20)	1	0.96 (0.92, 1.00)	1.11 (1.00, 1.24)	
≥65	N. of case	392	4,280	1,535	273	
	HR (95%CI)	1.16 (1.05, 1.30)	1	0.96 (0.90, 1.02)	1.08 (0.95, 1.23)	
Mortality of CVD						0.26
<45	N. of case	43	405	87	12	
	HR (95%CI)	1.17 (0.85, 1.62)	1	1.19 (0.93, 1.50)	1.39 (0.77, 2.50)	
45~65	N. of case	231	2,646	772	111	
	HR (95%CI)	1.03 (0.90, 1.19)	1	1.06 (0.97, 1.15)	1.50 (1.23, 1.83)	
≥65	N. of case	133	1,305	479	80	

	Early adulthood BMI (kg/m^2)				$P_{interaction}$
	<18.5	18.5~23.9	24.0~27.9	≥28.0	
HR (95%CI)	1.16 (0.97, 1.40)	1	1.01 (0.90, 1.13)	1.08 (0.84, 1.37)	

HRs were stratified by study regions and adjusted for sex, education, marital status, hypertension, family history, individual lifestyles, and weight change.

Table 8. Hazard ratios (95% confidence intervals) of mortality and morbidity of CVDs associated with each healthy lifestyle factor and the healthy lifestyle score.

	Lifestyle Factors [HR (95%CI)] [a]				
	Non-current daily smoker	Non-heavy alcohol drinker	Higher physical activity level	Higher diet quality score	One-point increase in healthy lifestyle score [b]
All-cause Mortality	0.73 (0.70, 0.75)	0.80 (0.78, 0.83)	0.85 (0.83, 0.88)	0.92 (0.83, 1.03)	0.80 (0.79, 0.82)
Mortality of Any CVD	0.72 (0.67, 0.77)	0.88 (0.82, 0.95)	0.84 (0.79, 0.89)	0.94 (0.76, 1.15)	0.82 (0.79, 0.84)
Morbidity of Any CVD	0.79 (0.78, 0.81)	0.92 (0.90, 0.95)	0.92 (0.90, 0.93)	0.91 (0.86, 0.96)	0.88 (0.87, 0.89)
Morbidity of IHD	0.77 (0.74, 0.79)	0.98 (0.95, 1.02)	0.90 (0.88, 0.92)	0.94 (0.87, 1.00)	0.88 (0.87, 0.90)
Morbidity of HS	0.87 (0.81, 0.93)	0.85 (0.79, 0.92)	0.92 (0.87, 0.98)	0.96 (0.76, 1.21)	0.89 (0.86, 0.92)
Morbidity of IS	0.82 (0.80, 0.85)	0.88 (0.85, 0.92)	0.93 (0.90, 0.95)	0.84 (0.78, 0.91)	0.88 (0.87, 0.90)

[a] The analyses were adjusted for sex, education, marital status, hypertension status, family history, early adulthood BMI, weight change, and all the individual lifestyle factors simultaneously.

[b] $P_{\text{linear trend}}$ <0.0001 for all endpoint (*P*=0.0002).

Table 9. Subgroup analysis of the association between early adulthood BMI and health outcomes (by midlife healthy lifestyle score).*.

		Early adulthood BMI (kg/m^2)			$P_{interaction}$
		<24.0	24.0~27.9	≥28.0	
**Morbidity of Any CVD**					0.18
0 or 1 healthy behaviour	No. of case	11,485	2,616	310	
	HR (95%CI)	1	1.15 (1.10, 1.21)	1.33 (1.19, 1.50)	
2 healthy behaviours	No. of case	22,270	5,140	631	
	HR (95%CI)	1	1.12 (1.09, 1.16)	1.45 (1.33, 1.57)	
3 or 4 healthy behaviours	No. of case	11,471	2,946	334	
	HR (95%CI)	1	1.15 (1.10, 1.20)	1.34 (1.20, 1.50)	
**Morbidity of IHD**					0.49
0 or 1 healthy behaviour	No. of case	5,767	1,313	165	
	HR (95%CI)	1	1.21 (1.13, 1.29)	1.47 (1.25, 1.72)	
2 healthy behaviours	No. of case	11,789	2,703	317	
	HR (95%CI)	1	1.14 (1.09, 1.19)	1.40 (1.25, 1.57)	
3 or 4 healthy behaviours	No. of case	5,996	1,485	183	
	HR (95%CI)	1	1.18 (1.11, 1.26)	1.54 (1.32, 1.79)	
**Morbidity of HS**					0.42
0 or 1 healthy behaviour	No. of case	1,473	404	46	
	HR (95%CI)	1	1.12 (0.99, 1.26)	1.12 (0.82, 1.52)	
2 healthy behaviours	No. of case	1,993	525	86	
	HR (95%CI)	1	1.05 (0.95, 1.17)	1.47 (1.17, 1.84)	
3 or 4 healthy behaviours	No. of case	1,091	321	39	
	HR (95%CI)	1	1.04 (0.91, 1.19)	1.03 (0.74, 1.44)	
**Morbidity of IS**					0.78
0 or 1 healthy behaviour	No. of case	6,126	1,353	157	
	HR (95%CI)	1	1.11 (1.05, 1.19)	1.24 (1.05, 1.46)	
2 healthy behaviours	No. of case	11,916	2,807	328	
	HR (95%CI)	1	1.14 (1.10, 1.20)	1.40 (1.25, 1.57)	
3 or 4 healthy behaviours	No. of case	5,815	1,533	157	
	HR (95%CI)	1	1.14 (1.08, 1.22)	1.20 (1.02, 1.42)	
**All-cause Mortality**					0.0006
0 or 1 healthy behaviour	No. of case	7,046	1,940	210	
	HR (95%CI)	1	0.99 (0.94, 1.05)	0.88 (0.76, 1.02)	
2 healthy behaviours	No. of case	8,251	2,001	323	
	HR (95%CI)	1	0.91 (0.86, 0.96)	1.21 (1.07, 1.36)	
3 or 4 healthy behaviours	No. of case	3,749	1,083	151	
	HR (95%CI)	1	1.00 (0.93, 1.08)	1.19 (1.00, 1.41)	
**Mortality of Any CVD**					0.078
0 or 1 healthy behaviour	No. of case	1,833	520	60	
	HR (95%CI)	1.00	1.10 (0.99, 1.22)	1.01 (0.77, 1.32)	
2 healthy behaviours	No. of case	2,115	548	99	
	HR (95%CI)	1.00	0.97 (0.87, 1.07)	1.41 (1.14, 1.75)	

3 or 4 healthy behaviours	No. of case	815	270	44
	HR (95%CI)	1.00	1.16 (1.00, 1.35)	1.52 (1.10, 2.10)

* The analyses were adjusted for sex, education, marital status, hypertension status, family history, and weight change. Multiplicative interaction was evaluated using likelihood ratio test by means of comparing regression models with and without cross-product terms.

Table 10. Subgroup analysis of the association between early adulthood BMI and health outcomes (by midlife smoking habits).*.

		Early adulthood BMI (kg/m^2)				$P_{interaction}$
		<18.5	18.5~23.9	24.0~27.9	≥28.0	
**Morbidity of CVD**						0.014
Unhealthy	N. of case	1,070	14,183	3,490	423	
	HR (95%CI)	1.03 (0.97, 1.10)	1	1.16 (1.12, 1.21)	1.45 (1.31, 1.60)	
Healthy	N. of case	3,383	26,590	7,212	852	
	HR (95%CI)	0.95 (0.92, 0.99)	1	1.12 (1.09, 1.15)	1.36 (1.27, 1.46)	
**Morbidity of IHD**						0.079
Unhealthy	N. of case	576	6,949	1,707	211	
	HR (95%CI)	1.05 (0.96, 1.14)	1	1.20 (1.14, 1.27)	1.47 (1.28, 1.70)	
Healthy	N. of case	1,878	14,149	3,794	454	
	HR (95%CI)	0.94 (0.90, 0.99)	1	1.15 (1.10, 1.19)	1.44 (1.30, 1.58)	
**Morbidity of HS**						0.43
Unhealthy	N. of case	136	1,835	534	69	
	HR (95%CI)	1.18 (0.99, 1.41)	1	1.11 (1.00, 1.23)	1.36 (1.06, 1.75)	
Healthy	N. of case	266	2,320	716	102	
	HR (95%CI)	1.06 (0.93, 1.21)	1	1.05 (0.97, 1.15)	1.18 (0.96, 1.46)	
**Morbidity of IS**						0.37
Unhealthy	N. of case	522	7,506	1,822	213	
	HR (95%CI)	0.95 (0.87, 1.04)	1	1.15 (1.09, 1.22)	1.37 (1.19, 1.58)	
Healthy	N. of case	1,760	14,069	3,871	429	
	HR (95%CI)	0.95 (0.90, 1.00)	1	1.13 (1.09, 1.17)	1.27 (1.15, 1.40)	
**All-cause mortality**						0.19
Unhealthy	N. of case	568	8,781	2,485	285	
	HR (95%CI)	1.17 (1.07, 1.28)	1	0.96 (0.92, 1.01)	0.95 (0.84, 1.08)	
Healthy	N. of case	991	8,706	2,539	399	
	HR (95%CI)	1.11 (1.03, 1.18)	1	0.97 (0.92, 1.01)	1.21 (1.09, 1.34)	
**Mortality of CVD**						0.81
Unhealthy	N. of case	158	2,233	649	90	
	HR (95%CI)	1.09 (0.92, 1.29)	1	1.04 (0.95, 1.15)	1.27 (1.02, 1.59)	
Healthy	N. of case	249	2,123	689	113	
	HR (95%CI)	1.08 (0.94, 1.23)	1	1.07 (0.97, 1.17)	1.29 (1.06, 1.58)	

HRs were stratified by study regions, baseline age groups and adjusted for sex, education, marital status, hypertension, family history, individual lifestyles, and weight change.

Table 11. Subgroup analysis of the association between early adulthood BMI and health outcomes (by midlife drinking habits).*.

		Early adulthood BMI (kg/m^2)				$P_{interaction}$
		<18.5	18.5~23.9	24.0~27.9	≥28.0	
**Morbidity of CVD**						0.86
Unhealthy	N. of case	402	5,345	1,411	177	
	HR (95%CI)	1.03 (0.93, 1.14)	1	1.11 (1.04, 1.18)	1.22 (1.04, 1.42)	
Healthy	N. of case	4,051	35,428	9,291	1,098	
	HR (95%CI)	0.97 (0.94, 1.00)	1	1.14 (1.11, 1.16)	1.41 (1.33, 1.50)	
**Morbidity of IHD**						0.48
Unhealthy	N. of case	216	2,545	690	93	
	HR (95%CI)	1.09 (0.94, 1.26)	1	1.18 (1.07, 1.29)	1.38 (1.11, 1.72)	
Healthy	N. of case	2,238	18,553	4,811	572	
	HR (95%CI)	0.96 (0.92, 1.00)	1	1.16 (1.12, 1.20)	1.45 (1.33, 1.58)	
**Morbidity of HS**						0.19
Unhealthy	N. of case	57	760	238	23	
	HR (95%CI)	1.21 (0.91, 1.59)	1	1.10 (0.94, 1.29)	0.85 (0.55, 1.31)	
Healthy	N. of case	345	3,395	1,012	148	
	HR (95%CI)	1.09 (0.97, 1.22)	1	1.07 (0.99, 1.15)	1.36 (1.15, 1.62)	
**Morbidity of IS**						0.26
Unhealthy	N. of case	197	2,873	709	88	
	HR (95%CI)	0.92 (0.79, 1.07)	1	1.06 (0.97, 1.15)	1.13 (0.91, 1.41)	
Healthy	N. of case	2,085	18,702	4,984	554	
	HR (95%CI)	0.95 (0.91, 1.00)	1	1.15 (1.11, 1.18)	1.33 (1.22, 1.45)	
**All-cause mortality**						0.17
Unhealthy	N. of case	227	3,682	1,172	135	
	HR (95%CI)	1.17 (1.02, 1.35)	1	0.96 (0.90, 1.03)	0.87 (0.73, 1.05)	
Healthy	N. of case	1,332	13,805	3,852	549	
	HR (95%CI)	1.13 (1.06, 1.20)	1	0.96 (0.93, 1.00)	1.16 (1.06, 1.26)	
**Mortality of CVD**						0.32
Unhealthy	N. of case	64	822	272	32	
	HR (95%CI)	1.21 (0.93, 1.57)	1	1.08 (0.93, 1.25)	0.92 (0.63, 1.34)	
Healthy	N. of case	343	3,534	1,066	171	
	HR (95%CI)	1.06 (0.94, 1.19)	1	1.05 (0.97, 1.13)	1.42 (1.21, 1.67)	

HRs were stratified by study regions, baseline age groups and adjusted for sex, education, marital status, hypertension, family history, individual lifestyles, and weight change.

Table 12. Subgroup analysis of the association between early adulthood BMI and health outcomes (by midlife physical activity habits).*

		Early adulthood BMI (kg/m^2)				$P_{interaction}$
		<18.5	18.5~23.9	24.0~27.9	≥28.0	
**Morbidity of CVD**						0.95
Unhealthy	N. of case	2,885	24,755	6,282	745	
	HR (95%CI)	0.98 (0.94, 1.02)	1	1.12 (1.09, 1.16)	1.37 (1.27, 1.48)	
Healthy	N. of case	1,568	16,018	4,420	530	
	HR (95%CI)	0.96 (0.91, 1.02)	1	1.15 (1.11, 1.19)	1.41 (1.29, 1.54)	
**Morbidity of IHD**						0.49
Unhealthy	N. of case	1,598	13,242	3,372	385	
	HR (95%CI)	0.97 (0.92, 1.02)	1	1.15 (1.10, 1.19)	1.35 (1.22, 1.50)	
Healthy	N. of case	856	7,856	2,129	280	
	HR (95%CI)	0.97 (0.90, 1.04)	1	1.18 (1.13, 1.25)	1.61 (1.42, 1.82)	
**Morbidity of HS**						0.68
Unhealthy	N. of case	251	2,303	679	101	
	HR (95%CI)	1.13 (0.99, 1.29)	1	1.10 (1.00, 1.20)	1.37 (1.12, 1.69)	
Healthy	N. of case	151	1,852	571	70	
	HR (95%CI)	1.06 (0.89, 1.26)	1	1.05 (0.95, 1.16)	1.13 (0.88, 1.45)	
**Morbidity of IS**						0.91
Unhealthy	N. of case	1,510	13,344	3,391	391	
	HR (95%CI)	0.95 (0.90, 1.01)	1	1.13 (1.08, 1.17)	1.32 (1.19, 1.46)	
Healthy	N. of case	772	8,231	2,302	251	
	HR (95%CI)	0.94 (0.87, 1.01)	1	1.14 (1.09, 1.20)	1.29 (1.13, 1.47)	
**All-cause mortality**						0.80
Unhealthy	N. of case	993	10,104	2,829	392	
	HR (95%CI)	1.14 (1.07, 1.22)	1	0.97 (0.93, 1.01)	1.06 (0.96, 1.18)	
Healthy	N. of case	566	7,383	2,195	292	
	HR (95%CI)	1.13 (1.04, 1.24)	1	0.96 (0.91, 1.01)	1.14 (1.01, 1.29)	
**Mortality of CVD**						0.18
Unhealthy	N. of case	281	2,720	823	115	
	HR (95%CI)	1.11 (0.98, 1.26)	1	1.07 (0.99, 1.16)	1.16 (0.96, 1.42)	
Healthy	N. of case	126	1,636	515	88	
	HR (95%CI)	1.02 (0.85, 1.24)	1	1.02 (0.92, 1.14)	1.58 (1.25, 1.98)	

HRs were stratified by study regions, baseline age groups and adjusted for sex, education, marital status, hypertension, family history, individual lifestyles, and weight change.

Table 13. Subgroup analysis of the association between early adulthood BMI and health outcomes (by midlife diet habits).*.

		Early adulthood BMI (kg/m^2)				$P_{interaction}$
		<18.5	18.5~23.9	24.0~27.9	≥28.0	
**Morbidity of CVD**						0.73
Unhealthy	N. of case	4,328	39,808	10,456	1,249	
	HR (95%CI)	0.97 (0.94, 1.01)	1	1.13 (1.11, 1.16)	1.38 (1.30, 1.47)	
Healthy	N. of case	125	965	246	26	
	HR (95%CI)	0.97 (0.80, 1.18)	1	1.15 (0.99, 1.34)	1.65 (1.10, 2.47)	
**Morbidity of IHD**						0.76
Unhealthy	N. of case	2,373	20,476	5,339	652	
	HR (95%CI)	0.97 (0.93, 1.01)	1	1.16 (1.13, 1.20)	1.45 (1.34, 1.58)	
Healthy	N. of case	81	622	162	13	
	HR (95%CI)	0.95 (0.75, 1.22)	1	1.17 (0.97, 1.41)	1.15 (0.65, 2.03)	
**Morbidity of HS**						0.046
Unhealthy	N. of case	400	4,105	1,231	166	
	HR (95%CI)	1.11 (1.00, 1.24)	1	1.07 (1.00, 1.15)	1.24 (1.06, 1.46)	
Healthy	N. of case	2	50	19	5	
	HR (95%CI)	0.39 (0.09, 1.65)	1	1.45 (0.81, 2.59)	4.57 (1.57, 13.31)	
**Morbidity of IS**						0.57
Unhealthy	N. of case	2,216	21,124	5,576	632	
	HR (95%CI)	0.95 (0.91, 0.99)	1	1.13 (1.10, 1.17)	1.31 (1.20, 1.42)	
Healthy	N. of case	66	451	117	10	
	HR (95%CI)	1.06 (0.81, 1.39)	1	1.25 (1.00, 1.56)	1.45 (0.76, 2.79)	
**All-cause mortality**						0.46
Unhealthy	N. of case	1,527	17,240	4,946	676	
	HR (95%CI)	1.14 (1.08, 1.20)	1	0.96 (0.93, 0.99)	1.10 (1.02, 1.19)	
Healthy	N. of case	32	247	78	8	
	HR (95%CI)	1.38 (0.93, 2.03)	1	1.10 (0.83, 1.46)	1.25 (0.59, 2.64)	
**Mortality of CVD**						0.81
Unhealthy	N. of case	401	4,294	1,315	200	
	HR (95%CI)	1.08 (0.98, 1.21)	1	1.05 (0.98, 1.12)	1.32 (1.14, 1.53)	
Healthy	N. of case	6	62	23	3	
	HR (95%CI)	1.02 (0.42, 2.45)	1	1.47 (0.85, 2.54)	2.40 (0.66, 8.71)	

HRs were stratified by study regions, baseline age groups and adjusted for sex, education, marital status, hypertension, family history, individual lifestyles, and weight change.

Table 14. Additive interaction between early adulthood BMI and lifestyle factors on health outcomes.

Health outcome	RERI (95%CI)	AP (95%CI)		
		early adulthood BMI	lifestyle score	interaction
Morbidity of Any CVD	0.016 (0.005, 0.028)	0.326 (0.286, 0.365)	0.600 (0.552, 0.648)	0.074 (0.025, 0.123)
Morbidity of IHD	0.019 (0.003,0.036)	0.350 (0.297,0.402)	0.569 (0.507,0.630)	0.082 (0.018,0.145)
Morbidity of HS	0.000 (-0.031,0.032)	0.272 (0.121,0.423)	0.726 (0.533,0.918)	0.002 (-0.182,0.186)
Morbidity of IS	0.012 (-0.004,0.028)	0.325 (0.269,0.381)	0.618 (0.550,0.687)	0.056 (-0.013,0.126)
All-cause Mortality	0.001 (-0.015,0.017)	-0.108 (-0.181,-0.035)	1.103 (0.999,1.206)	0.005 (-0.066,0.077)
Mortality of Any CVD	-0.013 (-0.045, 0.019)	0.173 (0.073, 0.273)	0.875 (0.736, 1.014)	-0.048 (-0.173, 0.077)

Regression model was stratified by study regions, baseline age groups and adjusted for sex, education, marital status, hypertension, family history, and weight change.

Additive interaction was evaluated using relative excess risk due to interaction (RERI) and attributable proportion (AP) with their 95%CIs between the lifestyle score (per one point decrease) and early adulthood BMI status (per 2.5 kg/m^2 increase).

Figure 1. Flowchart for inclusion and exclusion of the study sample*.

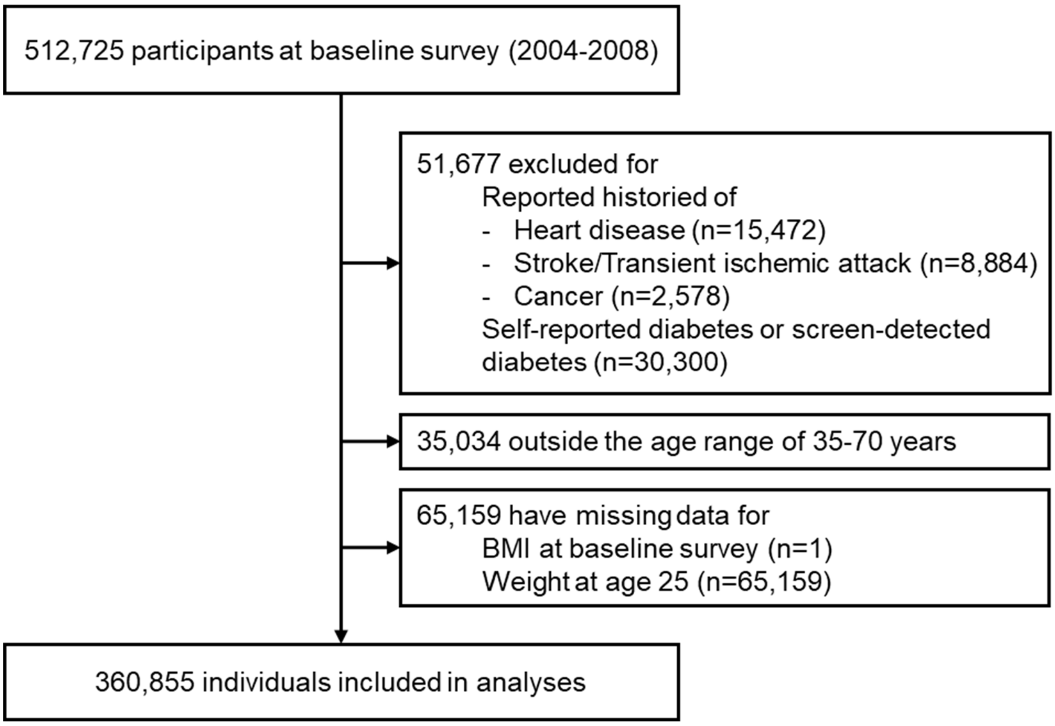

*Reasons for exclusion were not mutually exclusive, with 5558 participants meeting multiple exclusion criteria.

Figure 2. Association of early adulthood BMI with all-cause mortality and mortality of cardiovascular diseases (CVDs): restricted cubic spline (RCS).

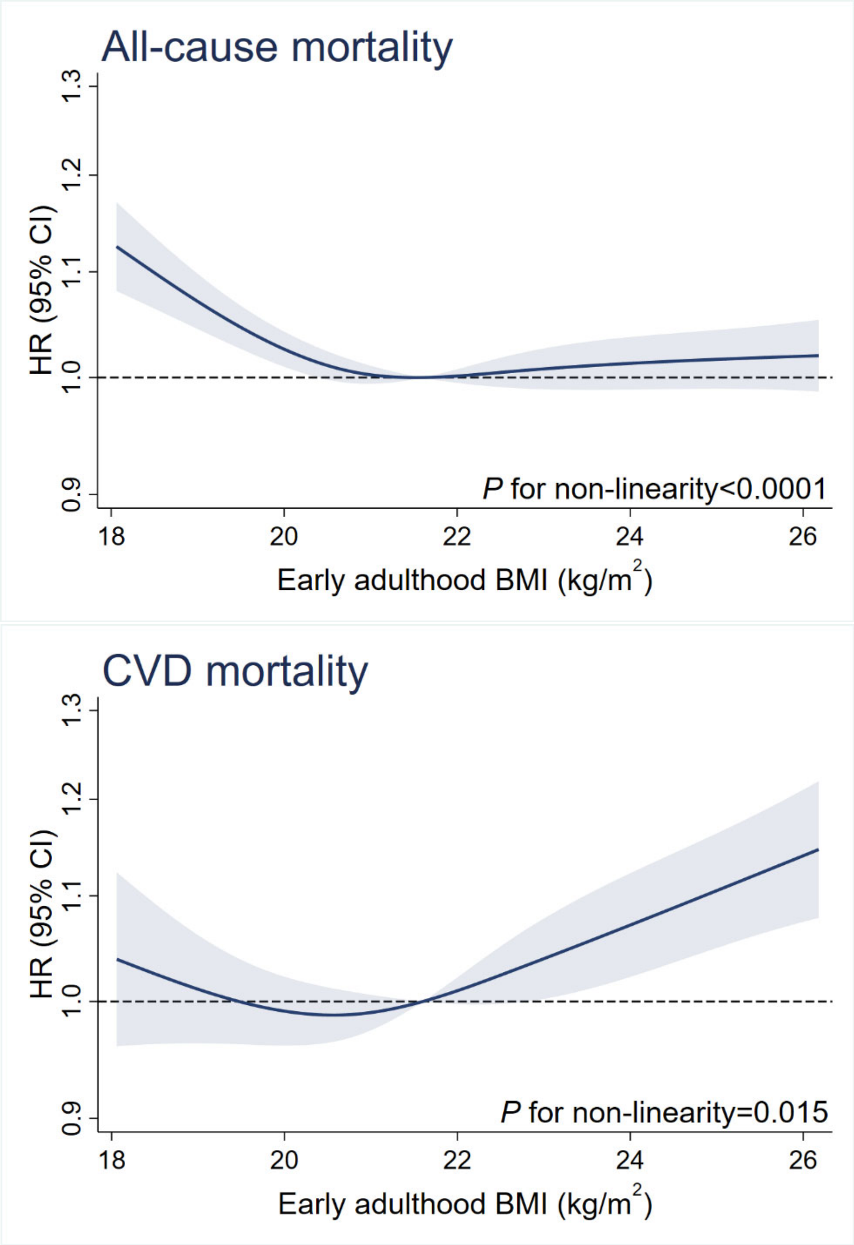

Estimates adjusted for sex, education, marital status, hypertension, family history, weight change and individual lifestyle factors, stratified by age and region.

Solid lines are multivariable adjusted hazard ratios, with area showing 95% confidence intervals derived from restricted cubic spline regressions with four knots. Reference lines for no association are indicated by the dashed lines at a hazard ratio of 1.0. The reference point is set as the median of the exposure (21.6 kg/m^2).

Figure 3. Attributable proportions from the early adulthood BMI, lifestyle score, and their interaction for incident CVDs*.

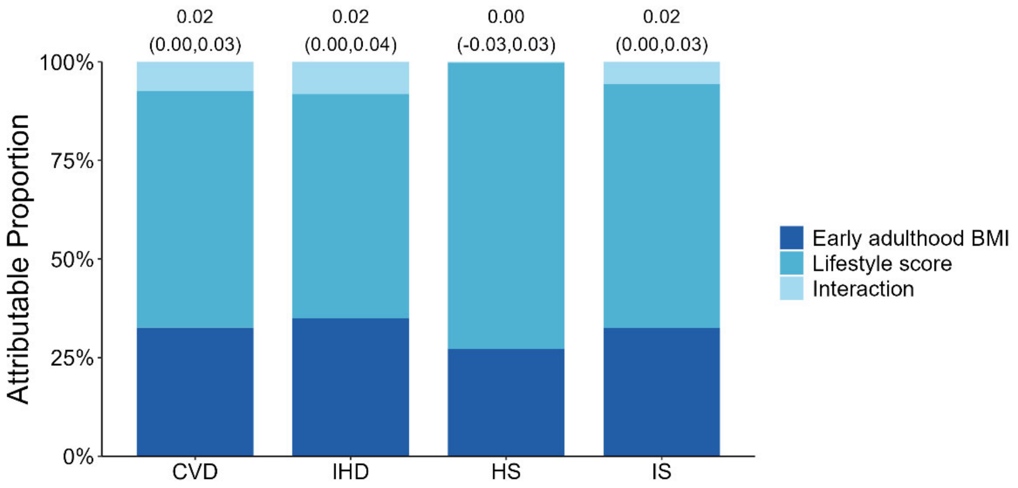

* The analyses were adjusted for sex, education, marital status, hypertension status, family history, and weight change. Number shows the point estimates and 95% confidence intervals for relative excess risk due to interaction (RERI) between early adulthood BMI and healthy lifestyle score.
